# Immune Checkpoint Inhibitor as a Fifth Component of Lipid Nanoparticles Enables Lymph Node‐Targeting and Augments mRNA Vaccine Efficacy

**DOI:** 10.1002/advs.77923

**Published:** 2026-09-27

**Authors:** Minki Ha, Jaehyun Kim, Seyoung Kim, Gonna Somu Naidu, Sungchan Cho, Seok‐Beom Yong

**Affiliations:** ^1^ Laboratory of Practical Nanomedicine Department of Medical and Biological Sciences The Catholic University of Korea Bucheon‐si Gyeonggi‐do Republic of Korea; ^2^ Department of Bioengineering Institute for Bioengineering and Biopharmaceutical Research Hanyang University Seoul Republic of Korea; ^3^ Nucleic Acid Therapeutics Research Center Korea Research Institute of Bioscience and Biotechnology (KRIBB) Chungcheongbuk‐do Republic of Korea; ^4^ Centre for Interdisciplinary Research SRM University‐AP Amaravati Andhra Pradesh India

**Keywords:** drug delivery, lipid nanoparticle, mRNA vaccine, PDL1 inhibitor‐incorporated LNP

## Abstract

mRNA‐encapsulated lipid nanoparticles (LNPs) have been used to develop vaccines and therapeutics since the COVID‐19 pandemic, and clinical studies have demonstrated therapeutic benefits of mRNA vaccines for various cancers. However, current LNP formulations still require functional modifications to tailor them for therapeutic and vaccine applications. Recently, fifth component‐incorporated LNPs were developed to finely tune their functionality, including immunogenicity and organ‐targeting ability. Here, biphenyl‐structured amphiphilic PDL1 inhibitors are employed as a fifth component to modify LNPs. Amphiphilic PDL1 inhibitor‐incorporated, ALC0315‐based LNPs show higher lymph node targeting, with a 6.94‐fold increase in mRNA expression in the inguinal lymph node, thereby improving lymph node expansion and immune cell infiltration and, consequently, inducing greater mRNA vaccine efficacy. Furthermore, a consistent lymph node‐targeting effect of the fifth component‐containing LNP was observed across studies using the SM102‐based LNP formulation, suggesting its universality. In conclusion, this study demonstrates that PDL1 inhibitor‐incorporated fifth‐component LNP serves as a next‐generation platform for mRNA vaccines.

## Graphical abstract

Here, a PDL1 inhibitor is employed as a fifth component to modify lipid nanoparticles (LNPs). PDL1 inhibitor‐loaded LNPs show enhanced lymph node targeting, resulting in greater lymph node expansion, immune cell infiltration, and improved mRNA vaccine efficacy. This fifth component LNP could be a novel platform for mRNA vaccines.

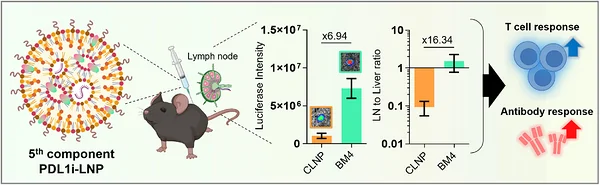

## 1 Introduction

Lipid nanoparticle‐encapsulated mRNA vaccines have been developed to treat and prevent various viral infections, including COVID‐19, RSV, and influenza [1, 2]. More recently, mRNA vaccines have demonstrated therapeutic effects across multiple cancer types, from triple‐negative breast cancer (TNBC) to pancreatic cancer, with durable T‐cell immune responses [3, 4, 5]. However, insufficient T‐cell responses and short‐lived antibody responses have been reported among recipients of mRNA vaccines [3, 6, 7]. In addition, vaccination efficacy varies depending on the immunological state of vaccinees, ranging from aging to disease state [8, 9, 10]. These studies suggest a need to further improve and optimize mRNA vaccines.

In recent decades, immune checkpoint signaling has been recognized as a key mechanism of immunosuppression in tumors, and inhibitors, such as antibodies against programmed cell death 1 (PD1), have been used to unleash anti‐tumor T cell immune responses [11]. PD1/PDL1 signaling is the most well‐established immune checkpoint pathway, and PDL1 on various cell types binds to PD1 on T cells to mediate inhibitory signaling. Specifically, myeloid cells, DCs, lymphatic endothelial cells, and cancer cells have been reported to express PDL1 [12, 13, 14]. Not only does it affect T cell responses, but it has also been revealed that PDL1 expression on regulatory B cells in the lymph nodes is highly involved in humoral immunity [15]. In addition, increased PDL1 expression has been reported in several diseases, including senescence [16, 17]. In a normal, healthy state, PD1/PDL1 signaling still functions as an immune checkpoint that regulates infection and inflammation; this is also relevant to vaccination‐induced immune responses [15]. Conclusively, these studies suggest that targeting PDL1 could be a novel strategy not only to target the vaccine‐relevant immune cells, such as DCs, but also to improve mRNA vaccine efficacy by unleashing T cell and B cell immune responses.

In recent studies, lipid nanoparticles were modified by the inclusion of fifth components, such as amphiphilic drugs and lipid‐drug conjugates, to improve their functionality, from targeting capability to immunogenicity modulation [18, 19, 20]. For instance, TLR agonist‐ and microbiome metabolite‐lipid conjugates were employed to improve mRNA vaccine efficacy [21, 22], and dexamethasone‐containing LNP reduced acute inflammatory responses [19]. Notably, vitamin D3 analog‐containing LNP was endogenously targeted to the pancreas with very high organ selectivity [23]. Because lymph nodes are key sites for mRNA vaccine‐induced immune responses, researchers have developed various ionizable lipid structures to improve LNP delivery to lymph nodes [24, 25, 26]. However, to the best of our knowledge, none of the studies have reported lymph node targeting by incorporating an amphiphilic drug into LNPs as the fifth component.

Here, lipid nanoparticles were modified with a small‐molecule inhibitor of PDL1 to enhance their functionality for mRNA vaccines. Biphenyl‐structured amphiphilic PDL1 inhibitors were co‐formulated in LNPs as the fifth component to develop PDL1 inhibitor‐incorporated LNPs (PDL1i‐LNP). BM4‐LNP, the outstanding candidate, shows significantly improved lymph node mRNA delivery and higher antigen‐specific T cell and antibody responses than clinically relevant conventional LNP formulations.

## 2 Results and Discussion

### 2.1 Physicochemical Characterization of PDL1 Inhibitor‐Incorporated Fifth Component LNPs

**FIGURE 1:**
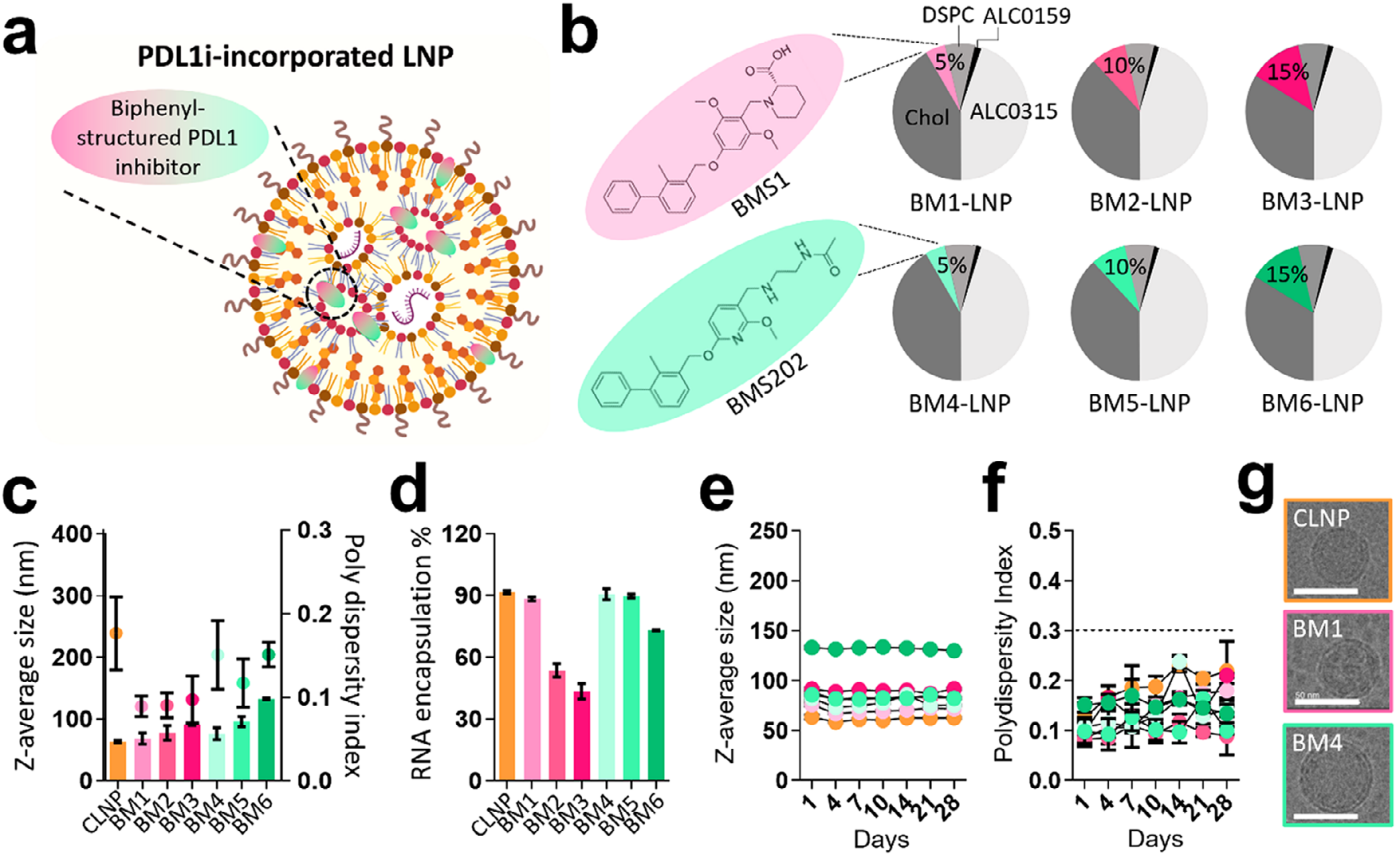
Scheme & physicochemical characterization of amphiphilic PDL1 inhibitor‐incorporated LNPs. (a) Schematic illustration of the biphenyl‐structured PDL1 inhibitor‐incorporated LNPs. (PDL1i‐LNPs) (b) Lipid molar composition of PDL1 inhibitor‐incorporated LNPs. All LNPs were prepared using ALC0315 ionizable lipid, DSPC, ALC0159, cholesterol (Chol), and firefly luciferase mRNA. Cholesterol was partially substituted with BMS‐1 and BMS‐202 at mol% levels ranging from 5% to 15% of the total lipid mol%. (c) DLS analysis of PDL1i‐LNPs. CLNP is conventional LNP formulation; 43.5:45:10:1.5(ALC0315: Chol: DSPC: ALC0159). The bar and circle in the graph indicate the Z‐average size and polydispersity index (PDI) of LNPs, respectively. (d) RNA encapsulation efficiency of LNPs. (e, f) Stability of PDL1i‐LNPs. The size (e) and PDI value (f) were measured at the indicated days post‐LNP preparation. Data are presented as mean ± S.E.M., *n* = 3–9 per group for ‘c‐d’, *n* = 3 per group for ‘e, f’. (g) Representative Cryo‐EM images of PDL1i‐LNPs. The Cryo‐EM images of CLNP, BM1‐LNP, and BM4‐LNP were shown. The white bar indicates 50 nm.

In previous studies, PDL1‐binding ligands, such as peptides, were employed as targeting moieties for tumor‐targeted mRNA and gene delivery [27, 28]. However, the ligand modification requires additional chemical conjugation to the LNPs' surface, and most previous studies applied PDL1 targeting in tumor therapy, not in mRNA vaccines. Partial substitution of cholesterol with the fifth component, such as amphiphilic drugs and lipid conjugates, endowed LNPs with novel functionality without adversely affecting RNA delivery efficiency [18, 19]. In this study, we employed biphenyl‐structured amphiphilic PDL1 inhibitors as a fifth component of LNPs, which have been used in cancer immunotherapy across various tumor models [29, 30]. BMS‐1 and BMS‐202 are amphiphilic PDL1 inhibitors with a biphenyl core structure at one end that binds and inhibits PDL1, and a hydrophilic moiety at the other. These PDL1 inhibitors were co‐formulated into LNPs to generate a small library of PDL1 inhibitor‐incorporated fifth‐component LNPs (PDL1i‐LNPs) (Figure 1a). ALC0315‐based Pfizer LNP formulation was employed, and the cholesterol composition of the LNP was partially substituted with BMS‐1 or BMS‐202, from 5–15% of total lipids, to develop PDL1i‐LNPs (Figure 1b). A total of 7 different LNPs encapsulating firefly luciferase mRNA were prepared using a microfluidic device. DLS results show a homogeneous size distribution across all PDL1i‐LNPs, with a modest increase in particle size observed upon increasing the proportion of the fifth component. Higher molar ratios of BMS‐1 or BMS‐202 increased LNP size and decreased RNA encapsulation (Figure 1c,d). More than 10% lipid mol substitution with BMS‐1 significantly reduced mRNA encapsulation efficiency to less than 60%, whereas BMS‐202 had a mild adverse effect on mRNA encapsulation efficiency. This difference may be attributable to structural differences in the hydrophilic region between BMS‐1 and BMS‐202. The ethylenediamine moiety of BMS‐202 is more hydrophilic than the piperidine carboxylic acid group of BMS‐1, which makes BMS‐202 more polar and suggests a high probability of incorporation into LNP. Stability analysis of mRNA‐encapsulated LNPs showed no significant differences in their size and PDI over 4 weeks post‐LNP preparation (Figure 1e,f). In addition, the integrity of the mRNAs was analyzed by measuring the concentration of LNP‐entrapped mRNAs. Compared to the BMS‐1‐loaded LNPs (BM1, BM2, and BM3), CLNP and BMS‐202‐loaded LNPs (BM4, BM5, and BM6) exhibited a higher integrity of LNP‐entrapped mRNA at 4 weeks post‐LNP preparation (Figure S1). The Cryo‐EM images show the spherical shape of conventional, BM1, and BM4 LNPs. A slightly enlarged morphology of BM4‐LNP was observed in comparison with conventional LNP (CLNP) (Figure 1g). There was a non‐significant difference in the surface charge of LNPs (Figure S2).

### 2.2 PDL1 Inhibitor‐Incorporated LNPs Enhance Lymph Node Delivery of mRNA

**FIGURE 2:**
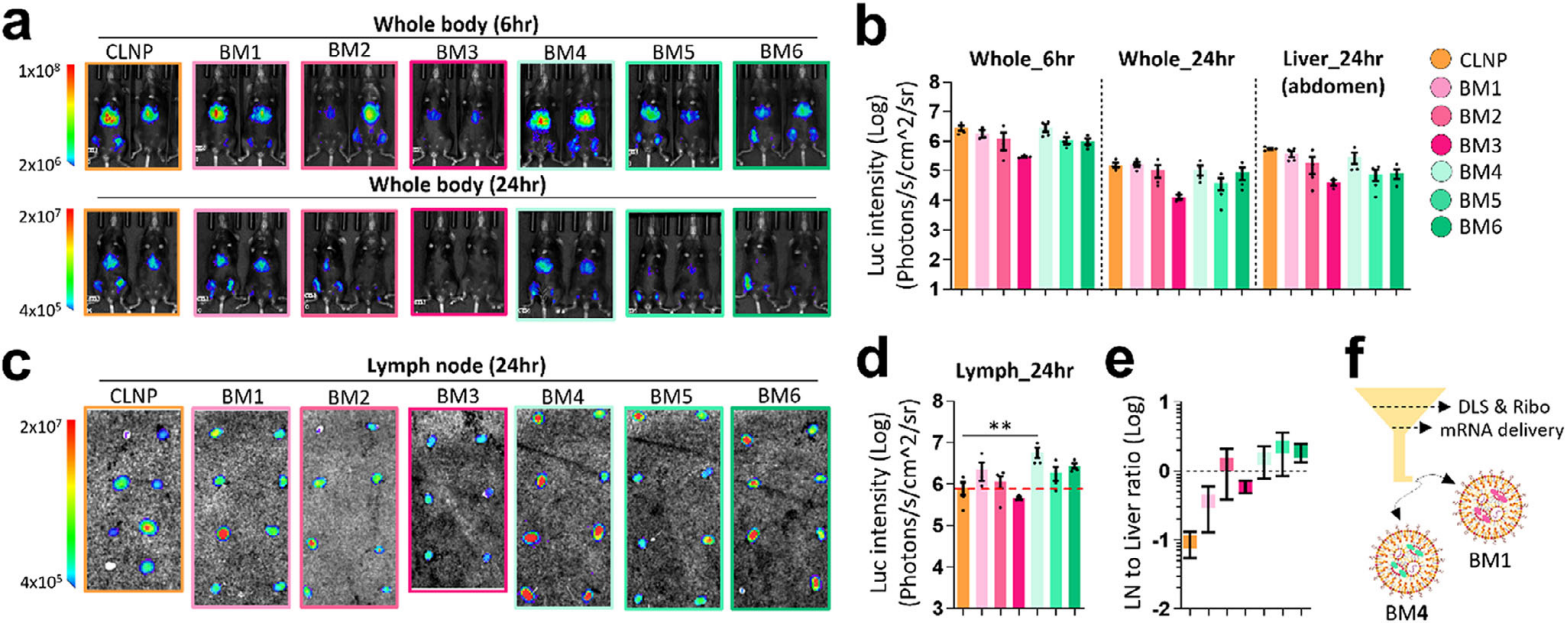
In vivo mRNA delivery efficiency & lymph node‐targeted delivery. (a) Representative images of in vivo mRNA delivery at the whole‐body level. Mice were injected with LNPs intramuscularly, and luciferase mRNA expression was measured at 6 and 24 h post‐injection. (b) Bar graph of the mRNA delivery efficiency in vivo. The bar graph shows the mean luciferase intensity at the whole‐body level. (c) Representative images of mRNA delivery for lymph nodes. Inguinal lymph nodes were harvested, and luciferase intensity was analyzed using IVIS. (d) Bar graph for the mRNA delivery efficiency in the inguinal lymph nodes. Each dot indicates the average luciferase intensity of two inguinal lymph nodes harvested from one mouse. (e) Relative ratios of luciferase mRNA expression in the lymph node to the liver. (f) Scheme for criteria for LNP screening. Data are presented as mean ± S.E.M., *n* = 3–4 mice per group. ^**^ *p* < 0.01 was analyzed by One‐way ANOVA with Tukey's post‐test.

To evaluate mRNA delivery efficiency in vivo, luciferase mRNA‐encapsulated LNPs were administered into the hind leg muscle in C57BL/6 mice. At 6 and 24 h post‐injection, luciferase intensity was measured at the whole‐body level, and the results showed decreased mRNA expression with increasing lipid mol substitution by PDL1 inhibitors compared with the conventional CLNP group. (Figure 2a,b). It has been reported that mRNA‐LNPs, upon intramuscular injection, traffic to adjacent lymph nodes and present antigens to stimulate immune cells, thereby inducing adaptive immunity [31]. Lymph node‐targeted mRNA delivery greatly improves mRNA vaccine efficacy [24]. In our lymph node mRNA expression results, a significantly higher lymph node‐targeting effect of the PDL1i‐LNPs than of the CLNP was observed (BM1 vs CLNP: 2.48‐fold; BM4 vs CLNP: 6.94‐fold) (Figure 2c,d). The luciferase expression analysis in the lymph node (LN) and liver (abdomen) indicates that all PDL1i‐LNPs have a higher LN‐to‐liver ratio than that of CLNP. Notably, 3.87‐fold and 16.34‐fold higher ratios were observed for BM1 and BM4 LNP groups, respectively, than for CLNP (Figure 2e). Otherwise, BM2, BM3, and BM5 LNPs did not significantly affect lymph node delivery. BM6 showed a lymph node delivery effect comparable to BM1 LNPs but had reduced mRNA encapsulation efficiency. Overall, BM1‐ and BM4‐ LNPs were chosen for further in vivo studies based on the results of physicochemical characterization and in vivo mRNA delivery (Figure 2f). In an additional experiment, we measured luciferase expression in major organs, including the liver, spleen, lungs, heart, kidneys, and bones, at 24 h post‐LNP injection. CLNP‐, BM1‐LNP‐, and BM4‐LNP‐injected groups exhibited comparable mRNA expression in the liver and spleen, with very weak mRNA expression in the bone (Figure S3). HPLC analysis was conducted to evaluate drug encapsulation in the LNPs. We measured the amount of PDL1 inhibitor in the dialyzed LNP sample, and the results show 57.12% and 71.03% drug encapsulation efficiency for BM1‐ and BM4‐LNPs, respectively. This result could be associated with the lower lymph node targeting effect of BM1‐LNP than that of BM4‐LNP (Figure S4).

### 2.3 PDL1 Inhibitor‐Incorporated LNP Enhances Immune Responses in the Lymph Node

**FIGURE 3:**
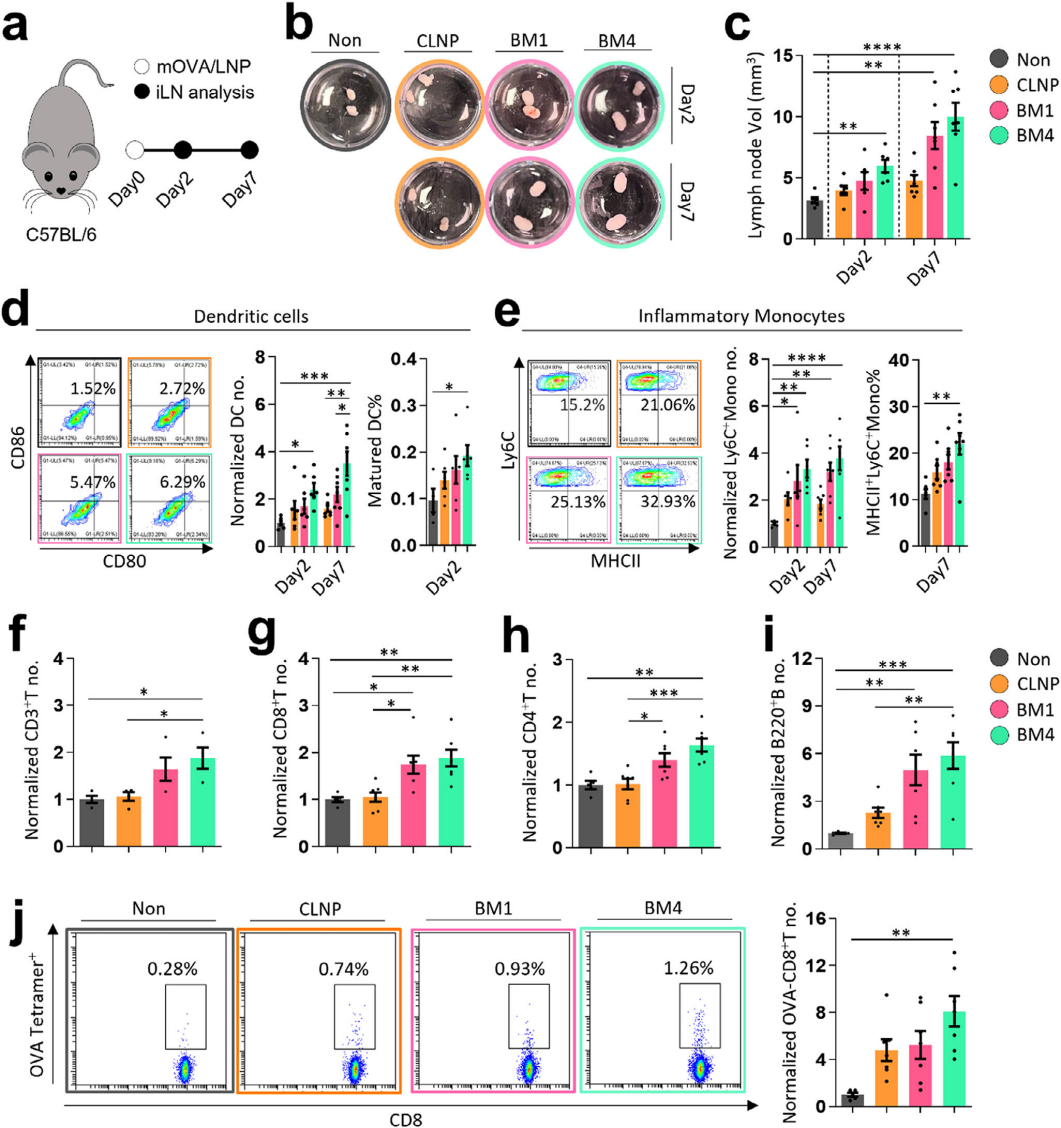
Improved immune responses of PDL1i‐LNPs in lymph nodes. (a) Experimental scheme. Mice were injected with chicken ovalbumin mRNA‐LNPs (mOVA/LNP) through the hind leg muscle, and inguinal lymph nodes (iLNs) were harvested to analyze the size and immune cell infiltration. (b) Representative images of inguinal lymph nodes. (c) Bar graph for the size of the lymph node. At 2 and 7 days post‐injection of mOVA/LNPs, inguinal lymph nodes were harvested to measure their volumes. (d) The phenotype of dendritic cells in lymph nodes. The number and maturation ratio of DCs (to CD45^+^ total immune cells) in inguinal lymph nodes were measured using flow cytometry at the indicated days. (e) The phenotype of inflammatory monocytes in lymph nodes. The number and ratio of MHC II expression in Ly6c^hi^ monocytes were measured using flow cytometry. Data are presented as mean ± S.E.M., *n* = 5 mice for non‐treated control and 6–7 mice per group for ‘c‐e’. ^*^ *p* < 0.05, ^**^ *p* < 0.01, ^***^ *p* < 0.001 were analyzed by One‐way ANOVA with Tukey's post‐test. (f–i) T and B cell analysis in inguinal lymph nodes. The numbers of CD3^+^(f), CD8^+^(g), CD4^+^(h), and B220^+^ B cells (i) were analyzed by flow cytometry at 7 days post‐injection. Cell counts were normalized to inguinal lymph node size to calculate relative cell numbers. (j) OVA antigen‐specific CD8^+^ T cell analysis in the lymph nodes. OVA antigen‐specific CD8^+^ T cells were detected using a fluorescent OVA antigen tetramer. Data are presented as mean ± S.E.M., *n* = 4 mice per group for ‘f’ and 6–7 mice per group for ‘g‐j’. ^*^ *p* < 0.05, ^**^ *p* < 0.01, ^***^ *p* < 0.001 were analyzed by One‐way ANOVA with Tukey's post‐test.

To evaluate immune responses in the lymph node, ovalbumin mRNA was encapsulated in LNPs and injected into the hind leg muscle of mice (Figure 3a). Lymph node expansion and cellular reconstitution have been linked to vaccination efficacy [32]. At 2 and 7 days post‐LNP injection, the inguinal lymph node (iLN) expanded significantly in groups vaccinated with BM1‐ and BM4‐LNPs compared with the CLNP group (Figure 3b,c). We then analyzed immune cell reconstitution by flow cytometry (Figure S5). In accordance with the expansion of the lymph node, the number of DCs was greatly increased in the iLN of PDL1i‐LNP groups, and the BM4‐LNP group exhibited the highest ratio of DC maturation, which is marked by the co‐expression of CD80 and CD86 (Figure 3d). In a previous study [32], MHCII‐expressing inflammatory monocytes were identified as mediators of vaccination magnitude, and these cells were highly infiltrated in the iLN of the BM4‐LNP group (Figure 3e). Moreover, not only total CD3^+^ T cells but also CD8^+^ and CD4^+^ T cells were highly infiltrated into the iLN of the PDL1i‐LNP groups (Figure 3f–h). Notably, B cell numbers were greatly increased in the iLN of PDL1i‐LNP groups, more than 2‐fold higher than in the CLNP group (Figure 3i). In addition, a greater number of OVA antigen‐specific CD8^+^ T cells was detected in the iLN of the BM4‐LNP group (Figure 3j). There was a comparable effect of BM1‐ and BM4‐LNPs on immune cell infiltration in the lymph node, but a more pronounced effect was observed for antigen‐specific CD8^+^ T cell induction by the BM4‐LNP than by the BM1‐LNP.

Even the BM1‐ and BM4‐LNPs elicited increased immune responses in the lymph node, but it is unclear whether PDL1 blockade mediates the effect. In a previous study, treatment with biphenyl‐structured PDL1 inhibitors blocked and modestly reduced PDL1 expression on the cells [29]. Based on that study, we measured PDL1 expression on the cells in the lymph node at 24 h post‐LNP injection. The DCs and monocytes exhibited stronger PDL1 expression levels (higher MFI values) than those of CD11b^+^ myeloid cells. The DCs and monocytes, not the CD11b^+^ myeloid cells from the lymph node of the mice injected with BM4‐LNP, showed modestly lower PDL1 expression levels compared to the CLNP‐ and BM1‐LNP‐injected groups (Figure S6a). This result indicates that BM4‐LNP mediates blockade of PDL1 on PDL1^−hi^ cells, such as DCs and monocytes. PDL1 inhibition on DCs has been reported to increase their activation/maturation [33, 34]. We could observe increased maturation of DCs (CD86^+^CD80^+^) by BM4‐LNP in vivo (Figure 3d), but this result does not exclude that the increased CD86/CD80 expression on DCs in vivo by the BM4‐LNP is derived from the higher concentrations of mRNA/LNPs in the lymph node (due to the lymph node targeting effect), rather than from the PDL1 blockade. To this end, bone marrow‐derived DCs (BMDCs) were treated with mRNA/LNPs *ex vivo* at the same concentration of mRNA/LNPs. At 24 h post‐LNP treatment, BM4‐LNP‐treated BMDCs exhibited a significantly higher level of CD80/CD86 expression in comparison with that of CLNP treatment (Figure S6b). These results suggest that the enhanced immune responses to BM4‐LNP are at least partially mediated by PDL1 blockade on immune cells such as DCs. In addition, we performed flow cytometric analysis using EGFP‐expressing mRNA to identify the main cell type expressing mRNA from BM4‐LNP. The EGFP mRNA‐encapsulated LNPs (CLNP, BM4‐LNP) were injected through the hind leg muscle of C57BL/6 mice, and then the lymph nodes were harvested at 6 h to measure EGFP expression. Compared with the CLNP group, the BM4‐LNP group showed a higher ratio of EGFP^+^ cells in total CD45^+^ immune cells and CD11c^+^MHCII^+^ DCs (Figure S7). However, we observed no significant difference in the ratios of EGFP^+^ cells among CD11b^+^ myeloid cells, which may be due to differences in PDL1 expression between DCs and myeloid cells (Figure S6a). Overall, BM4‐LNP exhibits higher lymph node delivery, PDL1 blockade, and immune cell activation than BM1‐LNP.

### 2.4 PDL1i‐LNP Improves Antigen‐Specific Immune Responses at the Systemic Level

**FIGURE 4:**
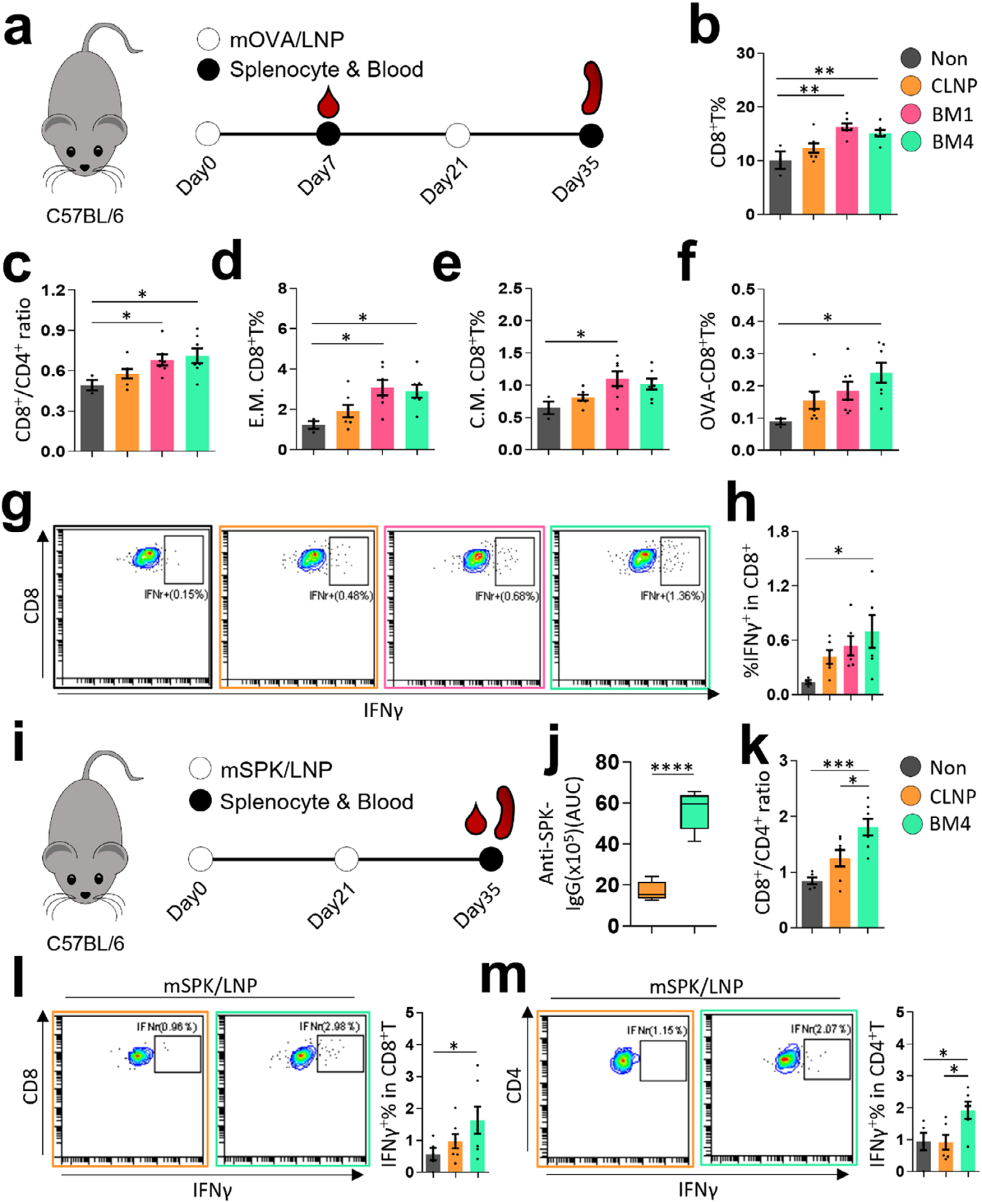
Antigen‐specific immune responses in blood and spleen. (a) Experimental scheme. Mice were intramuscularly injected with mOVA/LNP through the hind leg, and blood and splenocytes were harvested to analyze immune responses. (b–f). Blood T‐cell analysis. The ratios of CD8^+^ T cells (b), CD8/CD4 (c), effector memory CD8^+^T (d), central memory CD8^+^ T (e), and antigen‐specific CD8^+^ T cells (f) to CD45^+^ total immune cells were analyzed using flow cytometry. (g) Representative plots of the IFNγ^+^CD8^+^ T cells in splenocytes. Splenocytes were stimulated ex vivo with OVA peptide antigens for 20 h, and the ratio of IFNγ^+^ cells was then determined by flow cytometry. (h) Bar graph for the IFNγ^+^CD8^+^ T cell ratio. (i) Experimental scheme for the COVID‐19 spike mRNA vaccine study. Mice were vaccinated with COVID‐19 spike antigen mRNA‐encapsulated LNPs (mSPK/LNP) on days 0 and 21, and then splenocytes and serum were harvested at 35 days post‐LNP injection. (j). Anti‐SPK IgG levels in serum. Spike antigen‐specific IgG levels were measured, and the area under the curve (AUC) was shown. (k–m). Ex vivo analysis of splenocytes. The ratios of CD8/CD4 T cells (k), IFNγ^+^CD8^+^ T cells (l), and IFNγ^+^CD4^+^ T cells (m) were shown. Splenocytes were stimulated with a spike antigen peptide mixture for 20 h ex vivo and then analyzed by flow cytometry. Data are presented as mean ± S.E.M., *n* = 6–7 mice per group for ‘b‐m’. ^*^ *p* < 0.05, ^**^ *p* < 0.01, ^***^ *p* < 0.001 were analyzed by One‐way ANOVA with Tukey's post‐test.

To compare antigen‐specific immune responses at the systemic level, mOVA/LNPs were administered into mice (Figure 4a). At 7 days post‐mOVA/LNPs injection, BM1‐LNP induced a comparable CD8^+^ T cell response to that of BM4‐LNP in blood, and the BM4‐LNP group exhibited the highest antigen‐specific CD8^+^ T cell response, which is in line with the flow cytometric results in the lymph node (Figure 4b–f; Figure S8). This gap between the CD8^+^ T cell response and the antigen‐specific CD8^+^ T cell response observed in BM1 and BM4 LNPs could be explained by a previous study showing that the CD8^+^ T cell response does not necessarily reflect the antigen‐specific T cell response [35]. The result of ex vivo stimulation with OVA antigen peptides (SIINFEKL) exhibits a greater cytokine response in the splenocytes of the BM4‐LNP group than the CLNP group, with a 1.68‐fold increased ratio of IFNγ^+^ cells (Figure 4g,h; Figure S9). Furthermore, splenocytes from the BM4‐LNP group exhibited the highest levels of cytokine expression, including IL2 and TNFα (Figure S10). In the results of the COVID‐19 spike mRNA vaccination study (Figure 4i), the BM4‐LNP group exhibited more than 3‐fold greater AUC for anti‐Spike IgG titer in comparison with the CLNP group, which is in line with the higher B cell infiltration by BM4‐LNP shown in the flow cytometric result of the lymph node (Figure 4j). In addition, the increased humoral immune response of the BM4‐LNP group could be explained by the greater follicular helper T cells (FHT) and germinal center B cell (GCB) numbers in the lymph node of the BM4‐LNP group compared to the CLNP group (Figure S11). In splenic T cell responses, spike antigen peptide stimulation greatly increased the ratio of CD8^+^ to CD4^+^ T cells in the splenocytes of the BM4‐LNP group in comparison with the splenocytes of the non‐treatment and CLNP groups (Figure 4k). Also, splenocytes from the BM4‐LNP group exhibit a higher IFNγ response in the CD8^+^ and CD4^+^ T cells compared to that of the CLNP group by 1.68‐ and 2.1‐fold, respectively (Figure 4l,m).

### 2.5 Lymph Node‐Targeting and Vaccine‐Improving Effect of SM102‐Based PDL1i‐LNP

**FIGURE 5:**
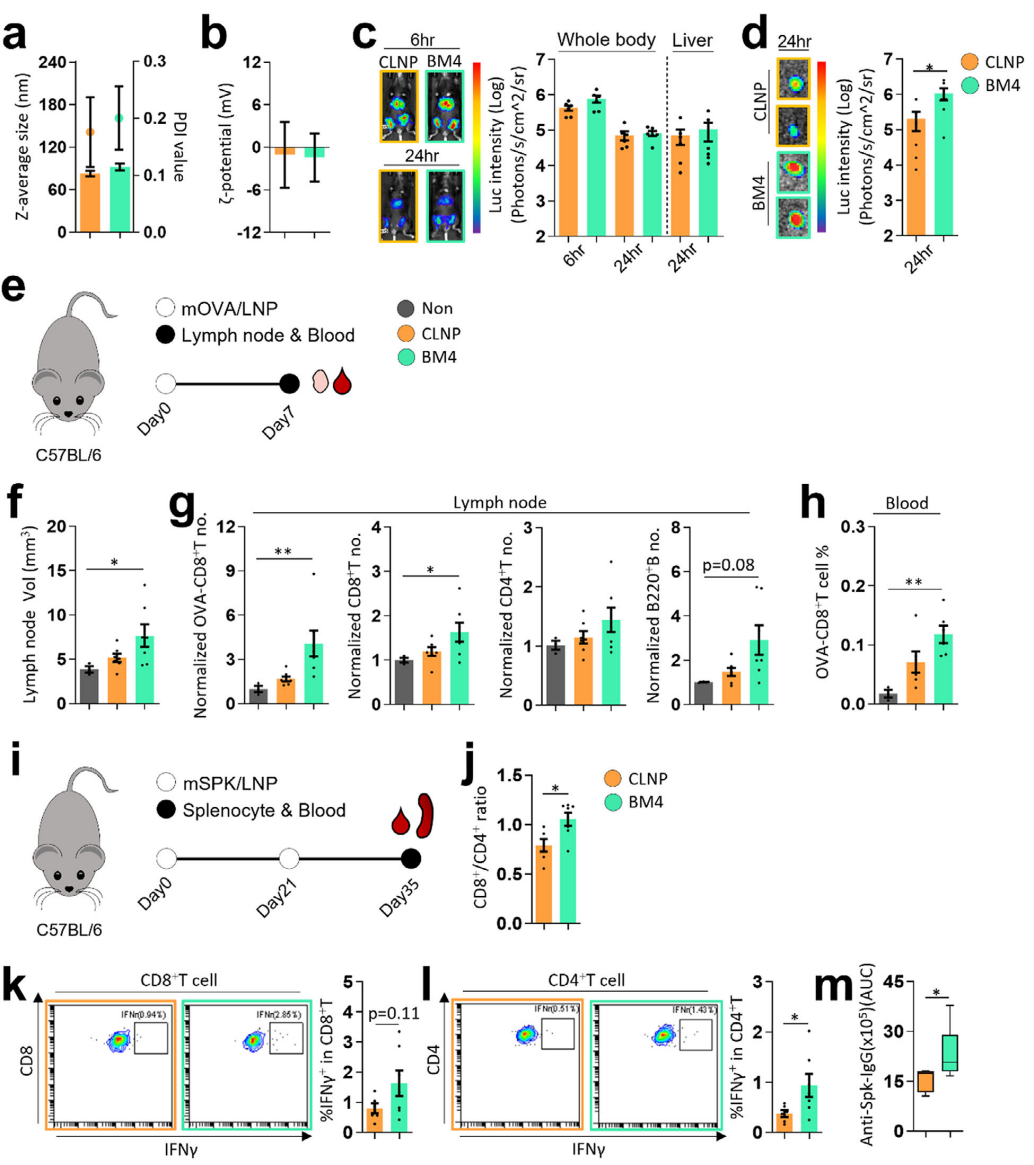
Lymph node‐targeting and immune‐improving effect of PDL1 inhibitor‐incorporated SM102‐LNP. (a, b). DLS analysis of SM102‐based LNPs. The SM102‐based CLNP and BM4‐LNP were prepared using a microfluidic device, and the size (a) and ζ‐potential (b) were analyzed by DLS assay. Data are presented as mean ± S.E.M., *n* = 6–9 per group. (c, d) In vivo mRNA delivery efficiency. Mice were intramuscularly injected with mLuc/LNPs through the hind leg, and then mRNA expression at the level of whole‐body & liver (c), and inguinal lymph node (d) was measured using IVIS. Representative images of mice and their inguinal lymph nodes, and a bar graph of luciferase intensity, were shown. (e) Experimental scheme of the OVA mRNA vaccine study. Mice were intramuscularly injected with mOVA/LNPs through the hind leg and sacrificed at 7 days post‐injection to harvest their organs. (f, g) The size of the lymph node and immune cell infiltrations. The volume (f) and the ratios of antigen‐specific CD8^+^ T cells and B220^+^ B cells (g) in the inguinal lymph node were shown. (h) The ratio of antigen‐specific CD8^+^ T cells in the blood. (i) Experimental scheme of the COVID‐19 Spike antigen mRNA vaccine study. Mice were intramuscularly vaccinated twice with mSPK/LNPs through the hind leg, and their splenocytes were harvested on day 35 post‐1st vaccination. (j–l) T cell responses in the COVID‐19 spike mRNA vaccine study. Splenocytes were stimulated with a spike antigen peptide mixture for 20 h ex vivo, and then the ratios of CD8/CD4 (j), IFNγ^+^CD8^+^ (k), and IFNγ^+^CD4^+^ (l) T cells were analyzed by flow cytometry. (m) Anti‐SPK IgG levels in serum. Spike antigen‐specific IgG levels were measured by ELISA and expressed as the area under the curve (AUC). Data are presented as mean ± S.E.M., *n* = 3 mice for non‐treat control and 6–7 mice per group for other groups in ‘c‐j’. ^*^ *p* < 0.05, ^**^ *p* < 0.01, ^***^ *p* < 0.001 were analyzed by the Mann‐Whitney test and Kruskal‐Wallis test.

To validate lymph node‐targeting and mRNA vaccine‐improving effects in another LNP formulation, SM102 LNP, Moderna's LNP formulation, was employed and modified via partial substitution of cholesterol with PDL1 inhibitor. As previously described in our results, BM4‐LNP (5% lipid mol substitution with BMS‐202) showed the highest lymph node‐targeting effect and higher vaccination efficacy than the conventional LNP formulation. Therefore, only the SM102‐based BM4‐LNP was prepared, and its mRNA vaccination efficacy was compared with that of the conventional SM102 LNP. DLS analysis shows a homogeneous size distribution of SM102‐based LNPs and a slightly larger size for BM4‐LNPs compared with CLNPs, consistent with the results for ALC0315 LNPs (Figure 5a). Also, no significant difference was observed in the ζ‐potential result (Figure 5b). Both CLNP and BM4‐LNP exhibited comparable in vivo mRNA delivery efficiency at the whole‐body level and in the liver (Figure 5c). BM4‐LNP exhibited significantly improved mRNA delivery in the inguinal lymph node, with a 5.24‐fold increase relative to CLNP (Figure 5d). The lymph node‐to‐liver luciferase intensity ratio increased 9.3‐fold, but this was not statistically significant (*p* = 0.065, Figure S12). The results of lymph node size, immune cell infiltration, and antigen‐specific CD8^+^ T cell responses in the OVA mRNA vaccination study indicate improved mRNA vaccine efficacy of BM4‐LNP in comparison with CLNP in the SM102 LNP formulation (Figure 5e–g). The results of the Spike mRNA vaccination study again reveal an improvement in antigen‐specific T cell responses with BM4‐LNP compared to CLNP (Figure 5h–i). Moreover, BM4‐LNP induced a higher magnitude of Spike antigen‐specific IgG than that of CLNP (Figure 5j). Compared to ALC0315‐based LNPs, SM102‐based LNPs induced a lower degree of lymph node expansion and B cell infiltration, and these are in line with the results of in vivo mRNA expression that exhibits several‐fold lower mRNA expression by the SM102‐based LNPs in comparison with ALC0315‐based LNPs (Figure S13).

### 2.6 Targeting Mechanism and In Vivo Toxicity Study

**FIGURE 6:**
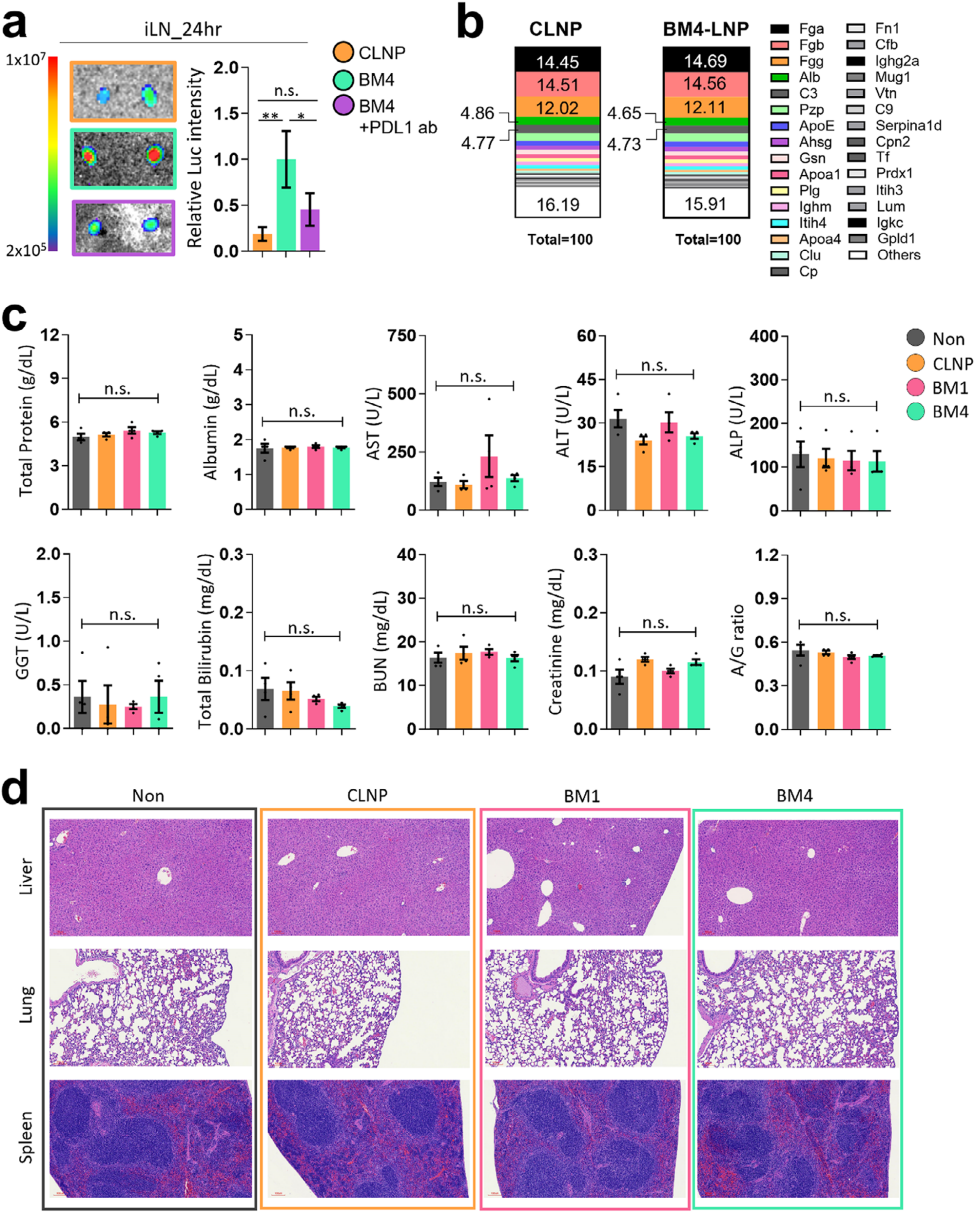
Targeting mechanism & in vivo toxicity analysis. (a) Effect of PDL1 blockade on lymph node delivery. Mice were pre‐treated with PDL1 antibody before the injection of BM4‐LNP. Data are presented as mean ± S.E.M., *n* = 7–9 mice per group. The ^*^ *p* < 0.05, ^**^ *p* < 0.01, ‘n.s.’(statistically non‐significant) were analyzed by Mann‐Whitney test. (b) Protein corona composition of LNPs. Plasma proteins adsorbed on the surface of LNPs were pooled and analyzed by LC‐MS/MS. The ratio of proteins to total coronal protein is shown as a fraction of the whole. *n* = 4 per group; ratios represent the mean of 4 replicates in the proteomic analysis. Fibrinogen alpha chain (Fga), fibrinogen beta chain (Fgb), fibrinogen gamma chain (Fgg), Albumin (Alb), Complement C3 (C3), Pregnancy zone protein (Pzp), Apolipoprotein E (ApoE), Alpha‐2‐HS‐glycoprotein (Ahsg), Isoform 2 of Gelsolin (Gsn), Apolipoprotein A‐I (Apoa1), Plasminogen (Plg), Isoform 2 of immunoglobulin heavy constant mu (Ighm), Inter alpha‐trypsin inhibitor, heavy chain 4 (Itih4), Apolipoprotein A‐IV (Apoa4), Clusterin (Clu), Ceruloplasmin (Cp), Fibronectin (Fn1), Complement factor B (Cfb), Ig gamma 2A chain C region secreted form (Ighg2a), Vitronectin (Vtn), Murinoglobulin‐1 (Mug1), Complement component C9 (C9), Carboxypeptidase N subunit 2 (Cpn2), Alpha‐1‐antitrypsin 1–4 (Serpina1d), Serotransferrin (Tf), Peroxiredoxin‐1 (Prdx1), Inter‐alpha‐trypsin inhibitor heavy chain H3 (Itih3), Immunoglobulin kappa constant (Igck), Lumican (Lum), Phosphatidylinositol‐glycan‐specific phospholipase D (Gpld1), Serum paraoxonase/arylesterase 1 (Pon1). (c) Blood chemistry analysis. Mice were intramuscularly injected with ALC0315‐based LNPs, and then the serum and major organs were harvested at 24 h post‐injection. The levels of total protein, albumin, Aspartate aminotransferase (AST), Alanine aminotransferase (ALT), Alkaline phosphatase, gamma‐glutamyl transpeptidase (GGT), Bilirubin, Blood urea nitrogen (BUN), creatinine, and albumin‐to‐globulin ratio (A/G ratio) were measured and shown. (d) Representative tissue histology images. The major organs, including the lung, spleen, and liver, were minced and H&E‐stained to evaluate PDL1i‐LNP toxicity. Data are presented as mean ± S.E.M., *n* = 4 mice per group. The ‘n.s.’ (statistically non‐significant) was analyzed by One‐way ANOVA with Tukey's post‐test.

Previously, several studies demonstrated endogenous targeting effects of LNPs to specific organs, such as the brain [20] and pancreas [23], and to specific immune cell types [36], and these effects were mediated by the structure of the lipid component rather than by ligand modification on the LNP surface. We hypothesized that the PDL1i‐LNP could endogenously target the lymph node via PDL1 targeting. To test this hypothesis, we treated mice with an anti‐PDL1 antibody before LNP injection and then measured mRNA expression in the lymph node. It is known that PDL1 is broadly expressed on immune cells in the lymph node, including dendritic cells (DCs) and myeloid cells, and indeed, we identified PDL1 expression on CD11c^+^ DCs, Ly6c^+^ inflammatory monocytes, and CD11b^+^ myeloid cells in the inguinal lymph node (Figures S6a and S14). Pre‐treatment with a PDL1 antibody reduced BM4‐LNP mRNA delivery by less than 45.5% compared with the non‐antibody‐blocked group, but it did not completely inhibit the lymph node mRNA expression. (Figure 6a). In addition, whole‐body expression levels did not differ significantly between groups (Figure S15).

The protein corona has been recognized as a mediator of LNP targeting to different organs, such as the lung and spleen [37]. To analyze the effect of the protein corona on lymph node targeting, plasma (C57BL/6) proteins bound to the LNPs were analyzed by LC‐MS/MS. As shown in the results, most of the proteins on the surface of BM4‐LNP were comparable to those on CLNP (Figure 6b). Only 8‐different coronal proteins exhibited statistically significant differences between the two LNPs, but the abundance of these proteins is extremely low (Figure S16, only less than 0.01% of total coronal proteins). Gas6 is one of the two proteins upregulated in the corona of BM4‐LNP and could mediate binding to immune cell receptors, such as MERTK [38]. Collectively, these results suggest that PDL1 binding, rather than alterations in protein corona composition, is the primary mechanism underlying the lymph node‐targeting effect of BM4‐LNP.

Finally, the toxicity of PDL1i‐LNPs was evaluated in vivo. At 24 h post‐LNP injection, blood chemistry was analyzed, and the results showed no significant differences in liver enzyme levels, total protein, or creatinine between the non‐treated and LNP‐treated groups (Figure 6c). Moreover, tissue histology images indicate that PDL1i‐LNP has no significant toxicity compared to conventional LNP in major organs such as the liver, spleen, and lung (Figure 6d). In addition, we analyzed the liver and spleen by flow cytometry to assess the effects of BM4‐LNP on immune cells. Both of the LNPs, CLNP and BM4‐LNP, infiltrated more CD45^+^ immune cells, CD11b^+^ myeloid cells, and Ly6c^+^ monocytes into the liver in comparison with the untreated control group, but the differences were not statistically significant (Figure S17a). Treatment with both LNPs also increased the ratio of DCs and Ly6c+ monocytes in the spleen, but not CD11b^+^ myeloid cells (Figure S17b). Compared with CLNP, BM4‐LNP treatment showed comparable effects on immune cells, except for splenic DCs, which had a higher ratio in BM4‐LNP‐treated mice.

As shown by the lymph node mRNA delivery and organ distribution results, BM4‐LNP significantly improved lymph node mRNA delivery but still accumulated highly in the liver and spleen. In previous studies [25, 26, 39], ionizable lipid structure‐dependent lymph node delivery strategies significantly altered the lymph node‐to‐liver ratio of mRNA delivery but only modestly increased mRNA delivery to the lymph node. In contrast, in our study, the fifth component strategy greatly enhances the absolute level of mRNA delivery to the lymph node.

In general, ionizable lipids constitute 30–50% mol ratio of the total lipids of the LNPs, which greatly affects the characteristics of the LNP. Collectively, we suggest that the fifth component strategy may preserve the intrinsic character of the LNPs, which results in a great increase in the ‘absolute level’ of lymph node mRNA delivery, whereas the ionizable lipid structure‐dependent strategy is more likely to change the intrinsic character of the LNPs, which consequently contributes to the ‘organ ratio’ of LNP delivery. Conclusively, this fifth‐component strategy is different from other previous studies from the point of view: (1) an amphiphilic drug as a fifth component of LNP; (2) increased absolute level of mRNA delivery to the lymph nodes; and lastly, (3) this fifth‐component strategy can be applied to various ionizable LNPs as a modular platform for lymph node mRNA delivery.

Repeated dosing of the immunogenic drugs could induce anti‐drug antibodies (ADAs). The drug's high molecular weight is a key factor in ADA induction, and small‐molecule drugs (e.g., BMS1: 475.58, BMS202: 419.52 g/mol) are generally unlikely to induce ADAs [40, 41, 42]. However, the immunogenicity of the small‐molecule inhibitor could affect ADA induction against LNPs. Overall, the potential for ADA induction against BM4‐LNP should be further investigated and compared with that of conventional LNPs in our future study.

For decades, PD1 and PDL1 signaling has been studied extensively in immunosuppressive diseases, mostly in cancers. Here, we validate the ‘proof‐of‐concept’ of PDL1i‐loaded LNPs for lymph node targeting and mRNA vaccine, and this system should be applied in various cancer models to evaluate its therapeutic effect as a cancer mRNA vaccine in our future study.

## 3 Conclusions

In this study, an amphiphilic PDL1 inhibitor is employed to modify LNPs to enhance lymph node‐targeting and mRNA vaccine efficacy, leveraging PDL1's inhibitory role in adaptive immunity. Biphenyl core‐structured BMS‐1 and BMS‐202 inhibitors were substituted in the composition of LNPs to develop a small library of LNPs, and the LNPs were screened through physicochemical characterization analysis and in vivo experiments. BM4‐ and BM1‐LNPs, with 5% molar substitution, showed enhanced lymph node‐targeting ability without adversely affecting mRNA delivery efficiency or particle stability. Specifically, BM4‐LNP showed the highest lymph node targeting of mRNA, with 6.94‐ and 5.24‐fold increases in the ALC0315‐ and SM102‐LNP formulations, respectively, suggesting the generalizability of our strategy. This targeting effect could be explained by the endogenous targeting effect of the LNPs, and it was substantially blocked by prior administration of an anti‐PDL1 antibody. The results of mRNA vaccination with OVA and Spike antigen demonstrate the mRNA vaccine‐improving effect of BM4‐LNP compared with conventional LNP formulations. Further investigation with SM102‐based LNPs again suggests that this strategy could be a modular platform to enhance lymph node‐targeted delivery and mRNA vaccination efficacy. In conclusion, we suggest an amphiphilic small‐molecule inhibitor of PDL1 as a fifth component of LNPs, and PDL1i‐LNP as a novel platform for next‐generation mRNA vaccines.

## 4 Materials and Methods

### 4.1 mRNAs

Chemically modified mRNAs, firefly luciferase (mLuc, 62 kD), chicken ovalbumin (mOVA, 43 kD), and enhanced green fluorescent protein (mEGFP, 27 kD) were purchased from TriLink.BioTechnologies.

### 4.2 In Vitro Transcribed RNA Preparation for COVID‐19 Spike mRNA

DNA templates for mRNA production were prepared using the gene synthesis service at Cosmogentech (T7 promoter + Pfizer COVID‐19 vaccine sequence). Using the BsmB1 restriction enzyme (NEB, R0739S), the DNA template (500 ng) was linearized, and the linearized DNA was purified. Based on a purified linear DNA template, COVID‐19 spike mRNA was produced with the HiScribe T7 High Yield RNA Synthesis Kit (NEB, E2040S) and components (5 mm ATP/CTP/GTP/N1‐methyl pseudoUTP, and 5 mm CleanCap Reagent AG (3’ OMe) [TriLink Biotechnologies, N‐7413‐10]) at 37°C for 2 h. After 2 h, the DNA template was removed using RQ1 DNase (RNase‐free [Promega, N6101]) and cleaned up using MEGAclear Transcription Clean‐Up Kit (Invitrogen, AM1908) to leave the COVID‐19 spike mRNA.

### 4.3 Lipid Nanoparticle Preparation

All ionizable lipids (ALC0315, SM‐102) and ALC0159 were purchased from BroadPharm (San Diego, CA, USA). DSPC, DMG‐PEG 2000, and Cholesterol were purchased from Avanti Polar Lipids Inc. BMS‐1 and BMS‐202 were purchased from MedChemExpress. All LNPs were prepared as described in a previous study [36]. In brief, 1 volume of lipids was prepared: Ionizable lipid, DSPC, PEG‐lipid, and Cholesterol at a 43.5:10:1.5:45 mol ratio in ethanol. SM102‐based LNP is composed of SM‐102, DSPC, DMG‐PEG2000, and Cholesterol. ALC0315‐based LNP is composed of ALC0315, DSPC, ALC0159, and Cholesterol. BMS‐1 and BMS‐202 were first dissolved in DMSO (30 mg mL^−^^1^) and then added to the other lipid mixture. Three volumes of mRNA (mLuc, mOVA, mSPK) in a sodium acetate buffer (pH 4.5) were mixed with 1 volume of lipids through a microfluidic mixing device, NanoAssemblr, IGNITE (Precision Nanosystems Inc), at a flow rate of 12 mL min^−1^. The prepared mRNA‐LNPs were dialyzed against phosphate‐buffered saline (PBS, pH 7.4) for 16–20 h, and PBS was refreshed twice. After dialysis of the mRNA/LNP, the LNP was stored at 4°C and used in experiments.

### 4.4 Lipid Nanoparticle Characterization

A Malvern Nano ZS ζ‐sizer (Malvern Instruments) was used to measure size and polydispersity index (PDI). mRNA/LNPs were diluted in phosphate‐buffered saline (PBS, pH 7.4) or distilled water (1:20, volume ratio) for size and PDI measurements. The zeta potential was measured using the same method as for size and PDI.

### 4.5 Quantification of mRNA Encapsulation

To measure mRNA encapsulation in LNPs, the Quant‐iT RiboGreen RNA assay kit (Thermo Fisher Scientific Inc.) was used. mRNA/LNPs were diluted 200‐fold with TE buffer (20 mM EDTA, 10 mm Tris‐HCl), and Triton X‐100 (0.5%, Sigma‐Aldrich)‐treated and non‐treated samples were prepared. A total of 100 µL of each sample was transferred to a 96‐well black plate (SPL), incubated at 37°C for 10 min, and then treated with 100 µL of TE buffer containing 0.5% RiboGreen reagent. The fluorescence values of mRNA in samples were detected using a Tecan microplate reader (TECAN).

$$\begin{array}{cc} & \mathrm{RNA}\;\mathrm{Encapsulation}\;\mathrm{efficiency}\;\left(\%\right)\\ & \;=\frac{\mathrm{RNA}\;\mathrm{conc}\;\left(w/\;\mathrm{Triton}\right)\;-\;\mathrm{RNA}\;\mathrm{conc}\;\left(w/o\;\mathrm{Triton}\right)}{\mathrm{RNA}\;\mathrm{conc}\;\left(w/\;\mathrm{Triton}\right)}\;\times 100 \end{array}$$

### 4.6 Animal Experiments

All animal protocols were approved by the Institutional Animal Care and Use Committee (IACUC) of The Catholic University of Korea and Korea Research Institute of Bioscience and Biotechnology (KRIBB). According to the relevant guidelines, the animal experiments were performed. Before the experiment, mice were randomly assigned.

### 4.7 In Vivo Luciferase Assay and Biodistribution Study

Six‐ to eight‐week‐old female C57BL/6 black mice (ORIENT Inc., Korea) were intramuscularly injected with mLuc/LNPs (mRNA dose: 3 µg per mouse, 1.5 µg per hind leg). At 6 and 24 h post‐LNP injection, mice were injected with D‐Luciferin (150 mg kg^−1^) through the peritoneal cavity. Whole‐body luciferase intensity was measured using the in vivo Optical Imaging System (IVIS, PerkinElmer, Inc.). For mRNA expression analysis in major organs, the heart, lungs, liver, spleen, kidneys, bones, and lymph nodes were harvested at 24 h post‐LNP injection. In the case of antibody blockade and mLuc/LNP administration, the anti‐PD‐L1 antibody was injected into mice via intramuscular injection (40 µg per mouse, 2 mg kg^−1^). After 1 h, mice were intramuscularly injected with mLuc/LNPs (mRNA dose: 3 µg per mouse, 1.5 µg per hind leg). Subsequently, in vivo luciferase intensity was measured using IVIS and compared with the non‐antibody‐blocked group.

### 4.8 In Vivo Immune Phenotype Study

mOVA/LNPs (mRNA dose: 3 µg per mouse, 1.5 µg per one hind leg) were intramuscularly injected into female C57BL/6 mice (ORIENT Inc., Korea). The mice were sacrificed at 2 and 7 days post‐LNP injection, and the inguinal lymph nodes were harvested for flow cytometric analysis. Lymph node volume was calculated using the same methods used for tumor measurement ([width^2^ × length]/2).

### 4.9 In Vivo EGFP Expression Study

Six‐ to eight‐week‐old female C57BL/6 black mice (ORIENT Inc., Kore) were intramuscularly injected with mEGFP/LNPs (mRNA dose: 3 µg per mouse), and the lymph nodes were harvested at 6 h post‐LNP injection. Lymph nodes were digested to a single‐cell suspension for further flow cytometric analysis.

### 4.10 Flow Cytometry Analysis of Immune Cells

Mice were sacrificed and organs were extracted from mice, homogenized, digested with Collagenase type IV (0.5 mg mL^−^^1^ in PBS), and filtered through a 100 µm cell strainer. Single cells were treated with red blood cell lysis buffer, incubated at room temperature for 5 min, and then centrifuged. The pellets were then washed twice with cold PBS. Cells were blocked with anti‐CD16/32 antibody for 20 min on ice and washed 2 times with FACS staining buffer (PBS supplemented with 1% FBS), and then stained with fluorescent antibodies. For flow cytometric analysis, the CytoFLEX and Cytexpert software (Beckman Coulter, USA) were used.

### 4.11 Vaccination and Adaptive Immune Responses Analysis

mOVA/LNPs and mSPK/LNPs were administered intramuscularly to female C57BL/6 mice on day 0(prime) and 21(boost) (mRNA dose: 3 µg per mouse, 1.5 µg per hind leg). Two weeks after the booster injection, the mice were sacrificed, and whole blood and spleen were collected. Blood samples were left at room temperature for 20 min, then centrifuged to separate serum. The antigen‐specific IgG in mouse serum was measured using ELISA kits (Mouse anti‐SARS‐CoV‐2 IgG titer serologic assay kit (ACRO Biosystems)). The area under the curve was measured using Prism software.

### 4.12 Ex Vivo Bone Marrow Dendritic Cell (BMDC) Activation Analysis

Bone marrow cells were harvested from six‐ to eight‐week‐old female C57BL/6 black mice (ORIENT Inc., Kore), and then seeded on a 24‐well tissue culture plate (10^6^ cells / well) in the presence of IL4 (25 ng mL^−^^1^) and GM‐CSF (25 ng mL^−^^1^) for BMDC differentiation. At 7 days of differentiation, cells were treated with mLuc/LNPs at an mRNA concentration of 1 µg mL^−^^1^. Cells were harvested 24 h after LNP treatment and analyzed by flow cytometry.

### 4.13 Antigen‐Specific T Cell Responses Analysis

Splenocytes were seeded (2 × 10^6^ per well) on a 24‐well tissue culture plate (Corning, Costar) and stimulated with an OVA peptide (SIINFEKL) or PepMix SARS‐CoV‐2 (JPT Peptide Technologies) at a concentration of 2 µg mL^−1^. After 20 h, the cells were washed twice with ice‐cold PBS and stained with antibodies for flow cytometric analysis.

### 4.14 Blood Chemistry and Tissue Histology Analysis

The female C57BL/6 mice were intramuscularly injected with mLuc/LNPs (mRNA dose: 3 µg per mouse, 1.5 µg per hind leg) for in vivo toxicity analysis. At 24 h post‐LNP injection, mice were sacrificed to harvest whole blood from the heart. Blood was centrifuged at 10,000 rpm to separate the serum. 10% neutral buffered formalin (BIOSESANG) was used to fix the organs for H&E staining and imaging. Blood chemistry analysis and tissue histology imaging were performed by CM Bio.

### 4.15 Cryo‐EM Imaging of LNPs

To prepare the cryo‐EM grid, 4 µL of LNP solution was applied to a Quantifoil holey carbon EM grid (R1.2/1.3, 300 mesh; EMS). Before applying the sample, the EM grids were glow‐discharged for 90 s at 15 mA. The grids were blotted with a Vitrobot Mark IV (FEI) for 3 s at 100% relative humidity at 4°C. Sample images were acquired using a Glacios (FEI) electron microscope equipped with a Falcon IV direct electron detector (FEI) at an acceleration voltage of 200 kV. Images were taken at 97,000× magnification, with a defocus of −2.0 µm.

### 4.16 HPLC Analysis of Drug Encapsulation Efficiency

PBS‐dialyzed LNP samples were freeze‐dried, and the powder was used to quantify drug content. The samples were dissolved in 30% MeOH, and the drugs were detected by HPLC on a C18 column (1260 Infinity II, Agilent) at 240 nm.

$$\begin{array}{cc} & \mathrm{Drug}\;\mathrm{Encapsulation}\;\mathrm{efficiency}\;\left(\%\right)\\ & \;=\frac{\mathrm{amount}\;\mathrm{of}\;\mathrm{the}\;\mathrm{drug}\;\mathrm{entrapped}\;\mathrm{in}\;\mathrm{LNPs}}{\mathrm{amount}\;\mathrm{of}\;\mathrm{the}\;\mathrm{drug}\;\mathrm{initially}\;\mathrm{added}\;\mathrm{to}\;\mathrm{prepare}\;\mathrm{LNP}}\;\times 100 \end{array}$$

### 4.17 Protein Corona Analysis

LNPs were prepared as described and dialyzed in PBS. Dialyzed LNPs were prepared to a lipid concentration of 3 mg mL^−^^1^. An equal volume of LNPs was mixed with C57BL/6 mouse plasma (C57BL/6 mouse plasma K2EDTA, BIOIVT) at a 1:1 volume ratio and incubated at 37°C for 20 min. Then, the LNPs were centrifuged at 15,000 rpm at 4°C for 10 min and washed 3 times with ice‐cold PBS. The pellets were used for proteomic analysis (LC‐MS/MS, Data‐independent acquisition method) by POCUS (SeoLin Bioscience Co. Ltd.).

### 4.18 Statistical Analysis

For statistical analysis, A Student's *t*‐test (Mann‐Whitney test) and one‐way ANOVA with Tukey's post hoc test and Kruskal‐Wallis’ test were used, and graphs and statistics were generated using GraphPad Prism 11 software. The data from this study are presented as mean ± standard deviation (S.D.) and standard error of the mean (S.E.M.). Pre‐established standards for removing animals from the experiments were based on animal behavior, health, and well‐being as required by ethical guidelines.

## Author Contributions

M. Ha, J. Kim, and S.‐B. Yong conceptualized and designed all experiments. M. Ha, J. Kim, and S.‐B. Yong performed physicochemical characterization of LNPs and in vivo and ex vivo experiments. The COVID‐19 Spike mRNA was synthesized by S. Kim and S. Cho. The first draft of the manuscript was written by M. Ha and S.‐B. Yong and edited by J. Kim, G. S. Naidu, and S. Cho. All authors have approved the final version of the manuscript.

## Conflicts of Interest

The authors declare no conflicts of interest.

## Supporting information




**Supporting File**: advs77923‐sup‐0001‐SuppMat.pdf.

## Data Availability

The data that support the findings of this study are available from the corresponding author upon reasonable request.
