# Supplementary material for: Immune Checkpoint Inhibitor as a Fifth Component of Lipid Nanoparticles Enables Lymph Node‐Targeting and Augments mRNA Vaccine Efficacy
Source: Adv Sci (Weinh). 2026 Sep 27:e77923. Online ahead of print. doi: 10.1002/advs.77923 (PMC13616246; doi:10.1002/advs.77923)
Supplement: Supplementary file 1 [file ADVS-9999-e77923-s001.pdf]

Supporting Information

Immune Checkpoint Inhibitor as a 5th Component of Lipid Nanoparticles Enables Lymph Node-Targeting and Augments mRNA Vaccine Efficacy

Minki Ha, Jaehyun Kim, Seyoung Kim, Gonna Somu Naidu, Sungchan Cho, Seok-Beom Yong**

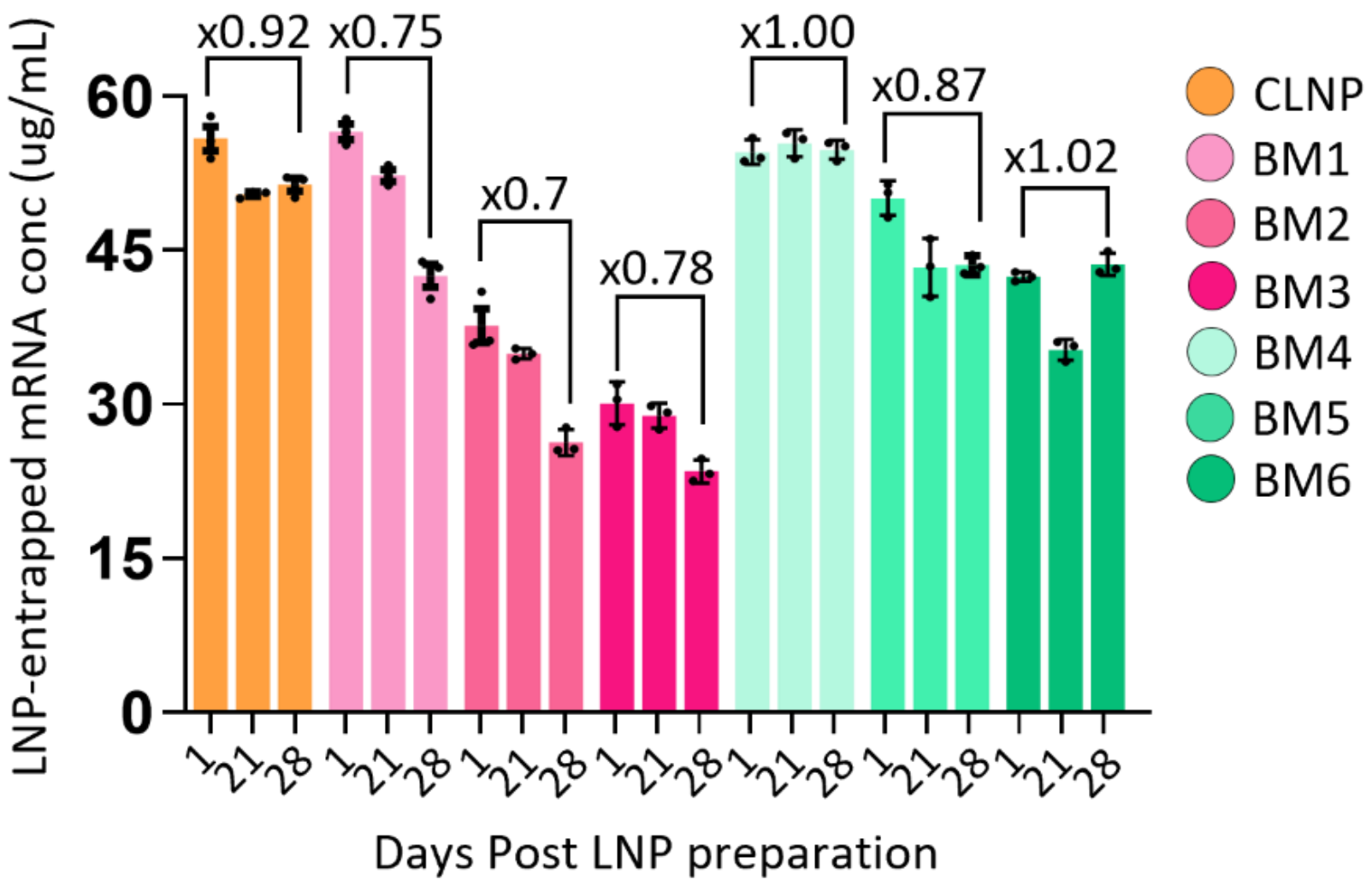

Supplementary Figure 1. Integrity of the LNP-entrapped mRNA. The concentration of the LNP-encapsulated mRNA was measured on the indicated days and compared to day 1. Data are presented as mean ± S.D., n=3 per group.

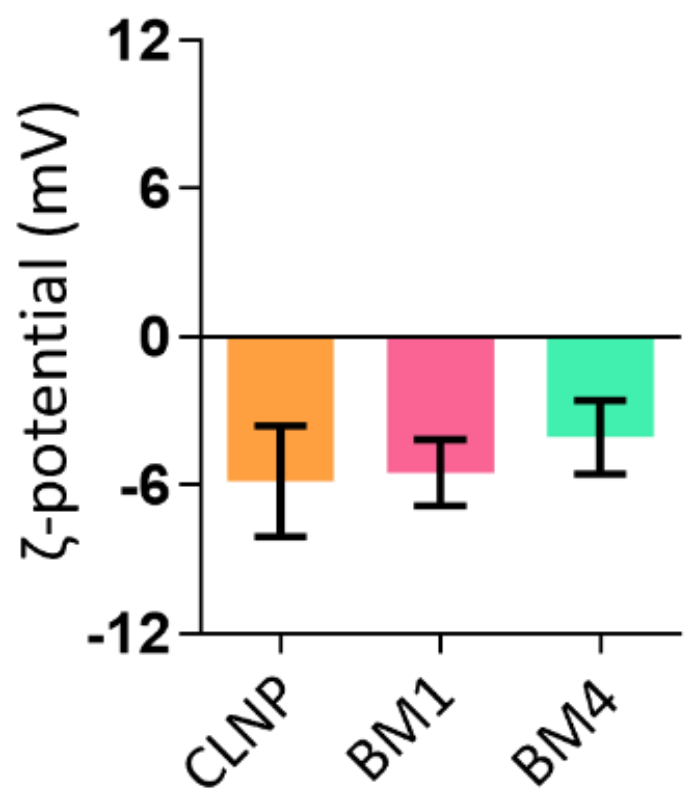

Supplementary Figure 2. ζ-potential of lipid nanoparticles. Surface charge of CLNP, BM1, and BM4-LNPs was analyzed by dynamic light scattering (DLS). Data is presented as mean ± S.D., n=6-9 per group.

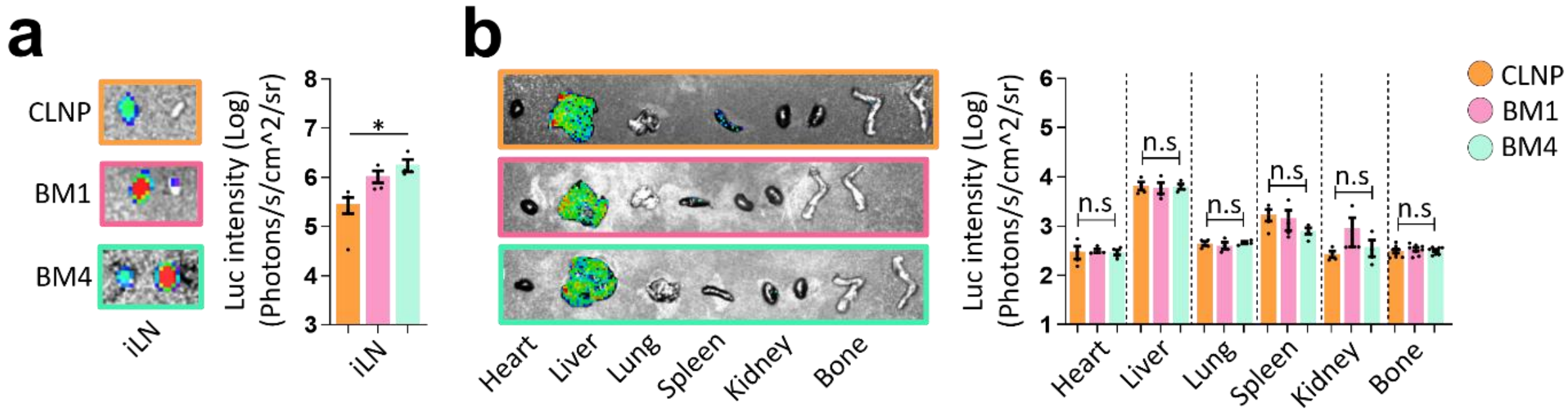

Supplementary Figure 3. Organ distribution of LNPs. Major organs were harvested at 24 hours post-mLuc/LNP injections, and luciferase expression was measured. **(a)** Luciferase expression in the inguinal lymph nodes. **(b)** Luciferase expression in the heart, liver, lungs, spleen, kidneys, and bones. Data are presented as mean ± S.E.M., mice n=4 per group. The **P*<0.05 and n.s. were analyzed by one-way ANOVA with Tukey's post-test.

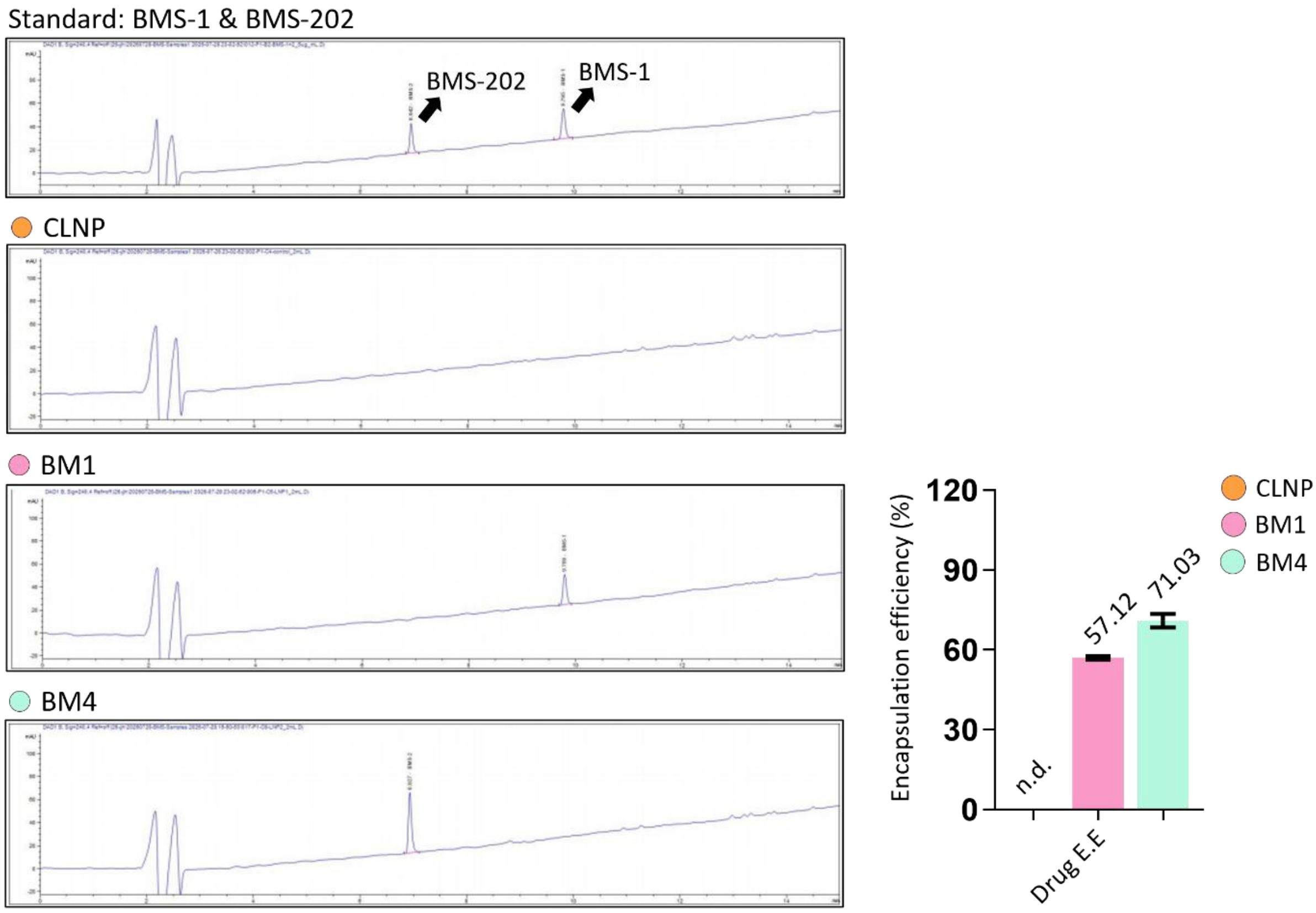

Supplementary Figure 4. Drug encapsulation analysis. Drug contents in the PBS-dialyzed BM1- and BM4-LNPs were analyzed by HPLC. The ratio of LNP-entrapped drug to the initially used drug was used to represent encapsulation efficiency (%). Data are presented as mean ± S.D. n=3 or 6 per group.

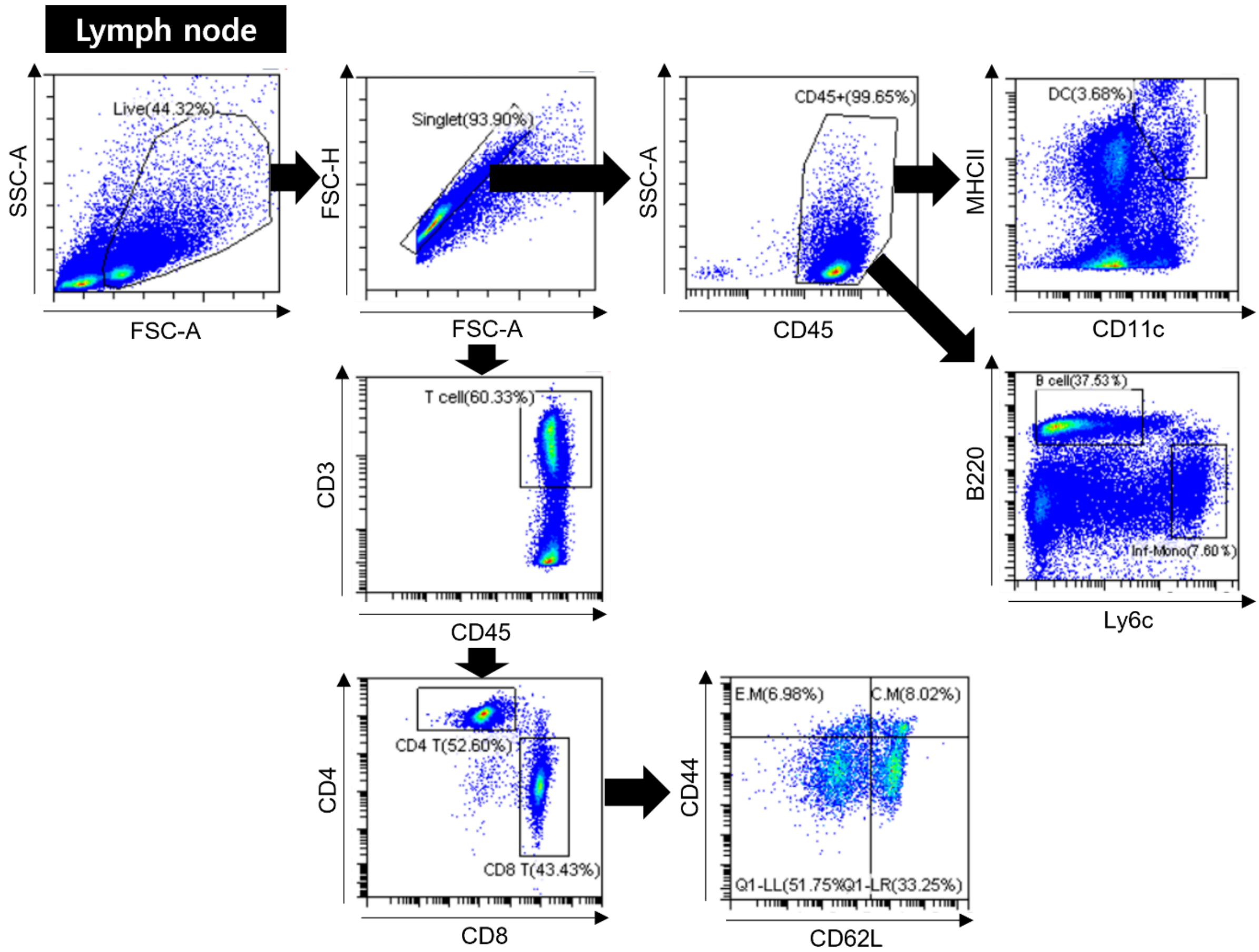

Supplementary Figure 5. Gating strategy for flowcytometric analysis of immune cells in the inguinal lymph node. Cells from the lymph node were stained with fluorescent antibodies and then analyzed by flow cytometry.

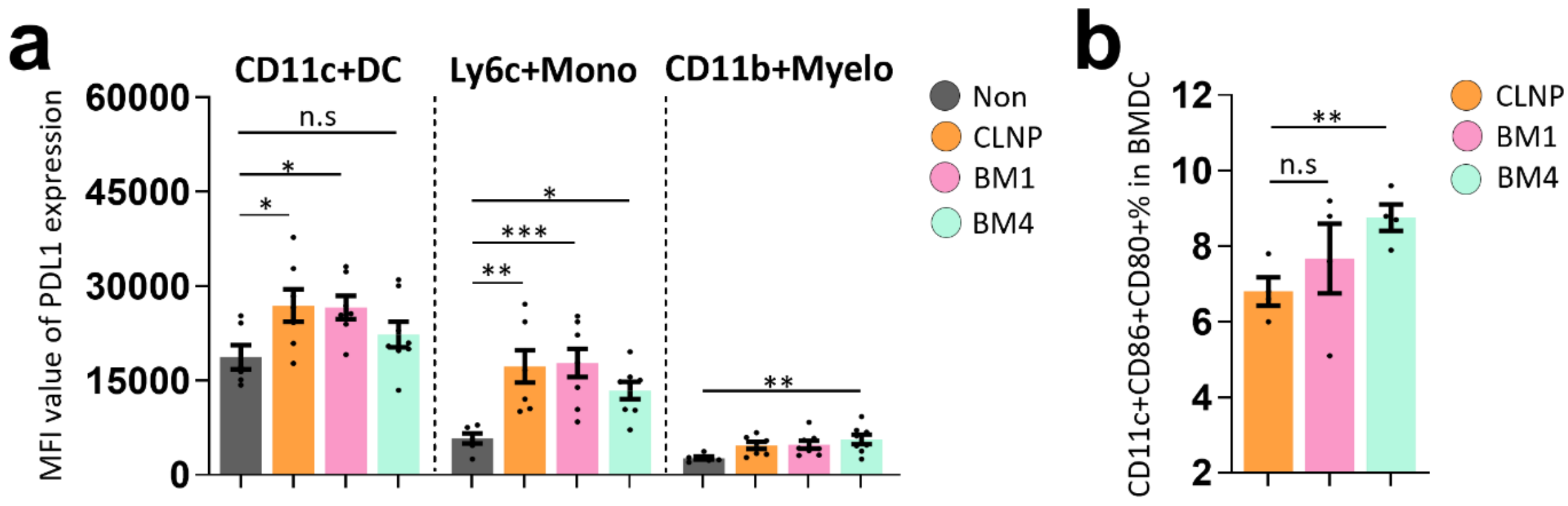

Supplementary Figure 6. *in vivo* PDL1 blockade & *ex vivo* DC activation analysis. (a) PDL1-expression on lymph node immune cells. At 24 hours post LNP-injection, PDL1 expression was evaluated by flow cytometry. Data are presented as mean ± S.E.M., mice n=3-4 per group (2 lymph nodes per mouse). The *P<0.05, **P<0.01, ***P<0.001, and n.s. were analyzed by one-way ANOVA with Dunnett's post-test. **(b)** The ratio of $CD11c^{+}CD86^{+}CD80^{+}$ cells in bone marrow-derived dendritic cells (BMDCs). Data are presented as mean ± S.E.M., n=4 per group. The **P<0.01 and n.s. were analyzed by the Mann-Whitney test.

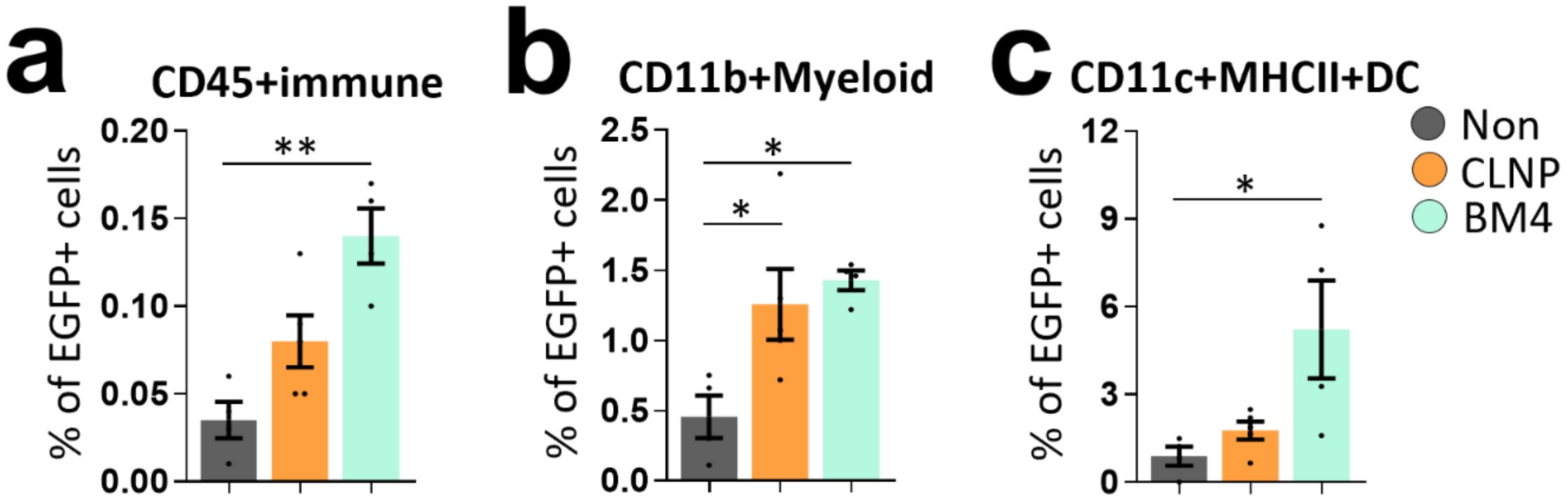

Supplementary Figure 7. Analysis of EGFP mRNA expression in the lymph node. At 6 hours post-LNP injection, lymph node immune cells were harvested and analyzed by flow cytometry to measure EGFP expression. The ratio of EGFP^{+} cells in CD45^{+} immune cells **(a)**, CD11b^{+} myeloid cells **(b)**, and CD11c^{+}MHCII^{+}DC **(c)** was shown. Data are presented as mean ± S.E.M., mice n=4-5 per group. The *P<0.05, **P<0.01 were analyzed by one-way ANOVA with Tukey's post-test.

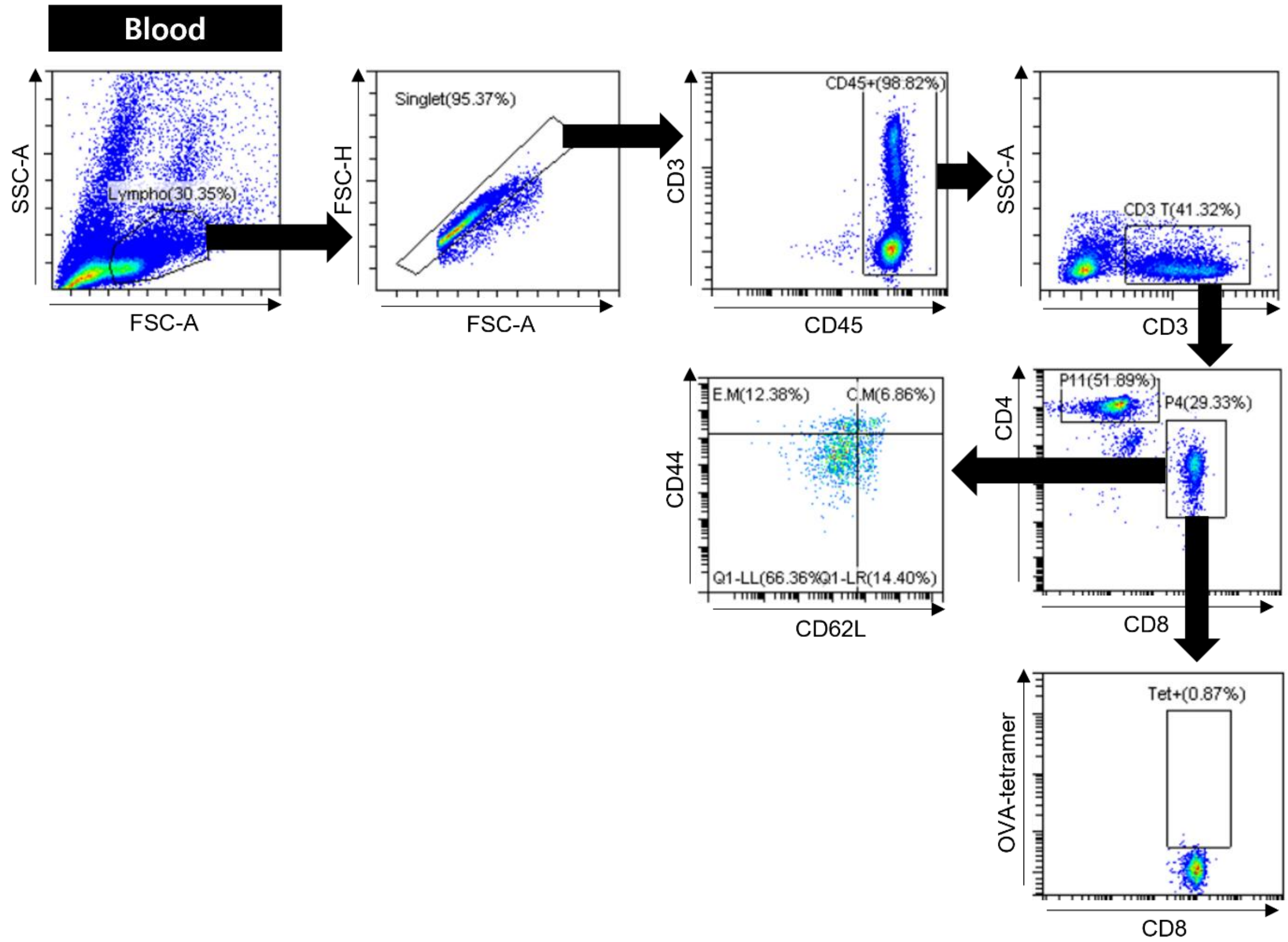

Supplementary Figure 8. Gating strategy for flowcytometric analysis of immune cells in the blood. RBC-lysed blood cells were stained with fluorescent antibodies and then analyzed by flow cytometry.

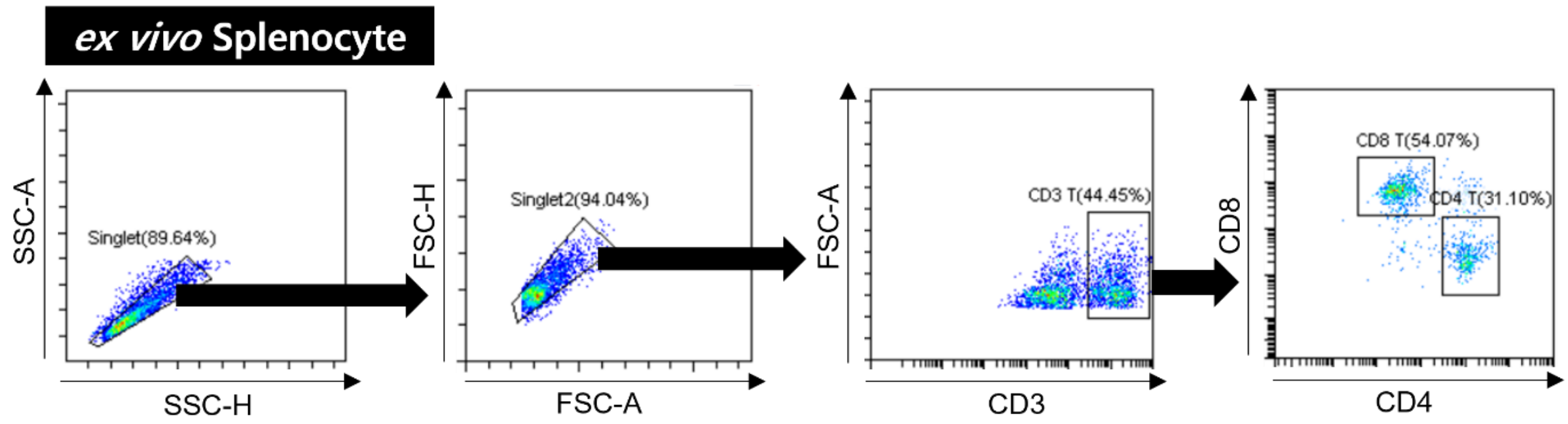

Supplementary Figure 9. Gating strategy for flow cytometric analysis of splenic T cells *ex vivo*. Peptide antigen-stimulated splenocytes were stained with fluorescent antibodies and then analyzed by flow cytometry.

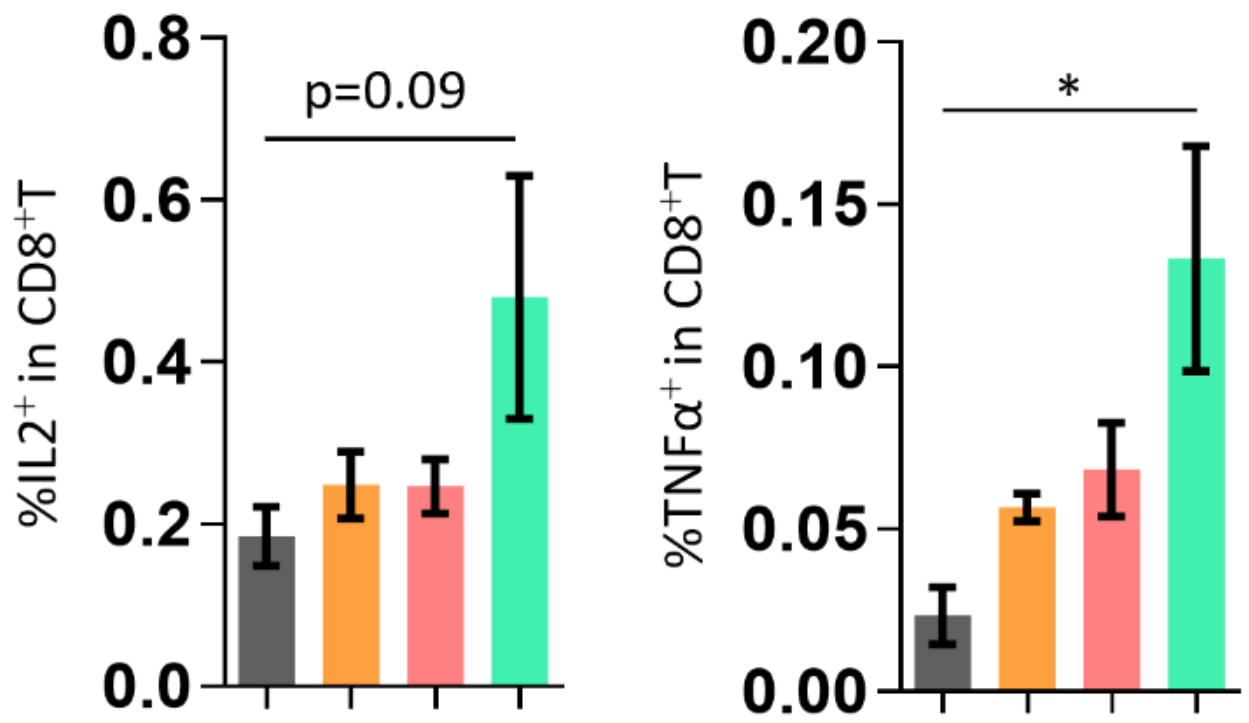

Supplementary Figure 10. Cytokine expression in CD8$^+$ T cells. Peptide antigen-stimulated splenocytes were stained with fluorescent antibodies for cytokine expression analysis (IL2, TNFα)—groups: Non-treat control, CLNP, BM1, and BM4. Data are presented as mean ± S.E.M., mice n= 4 for non-treat and n= 6 for other groups. *P*=0.09 and **P*<0.05 were analyzed by one-way ANOVA with Dunnett's post-test.

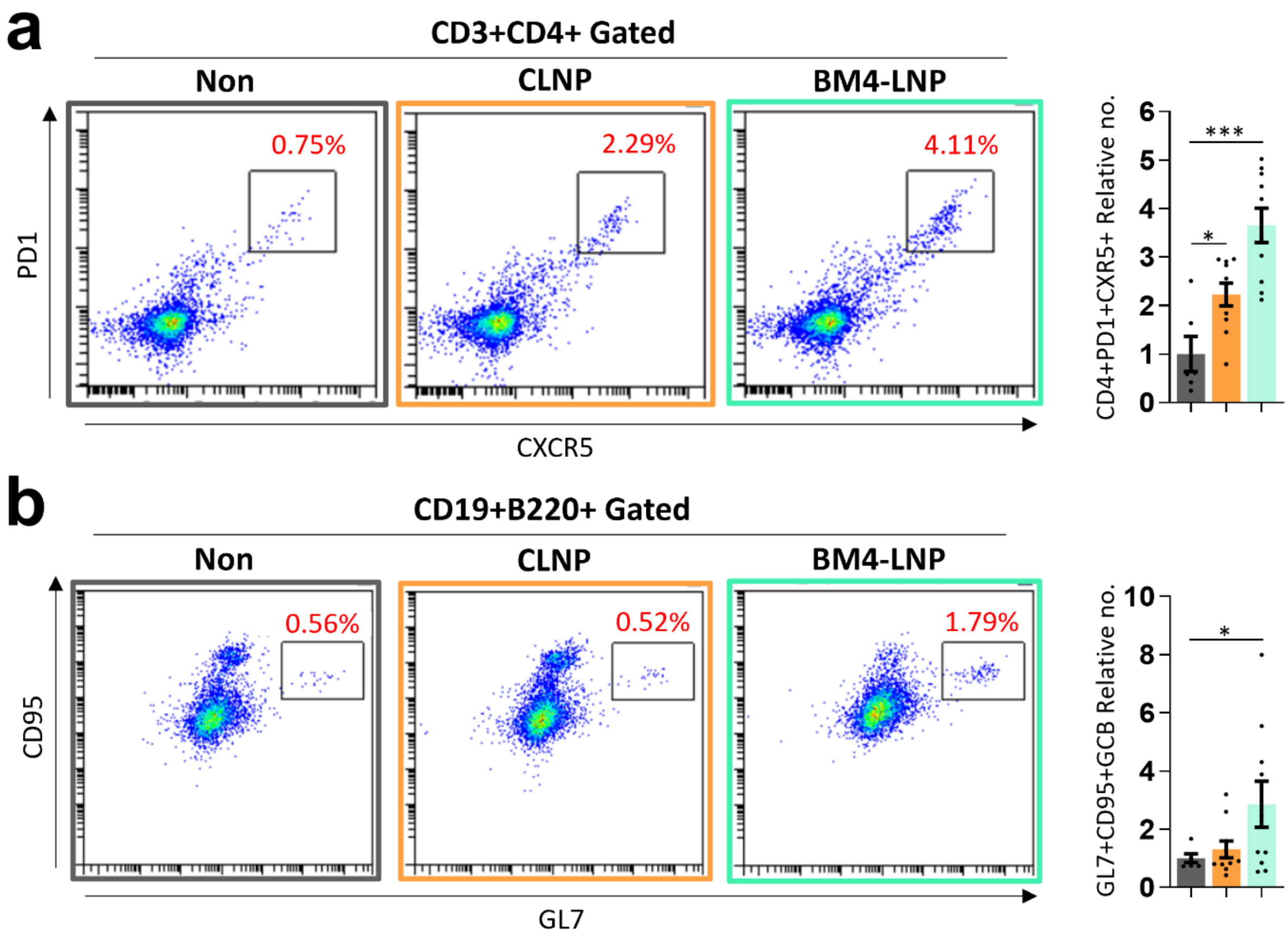

Supplementary Figure 11. Follicular helper T cells (FHT) & germinal center B cells (GCB) in the lymph nodes. At 7 days post-LNP injection, the ratios of FHT cells and GCB cells were analyzed in the lymph nodes and normalized to lymph node size. Data are presented as mean ± S.E.M., mice n = 4-5 per group (2 lymph nodes per mouse). The **P*<0.05, ****P*<0.001 were analyzed by the Welch's t-test.

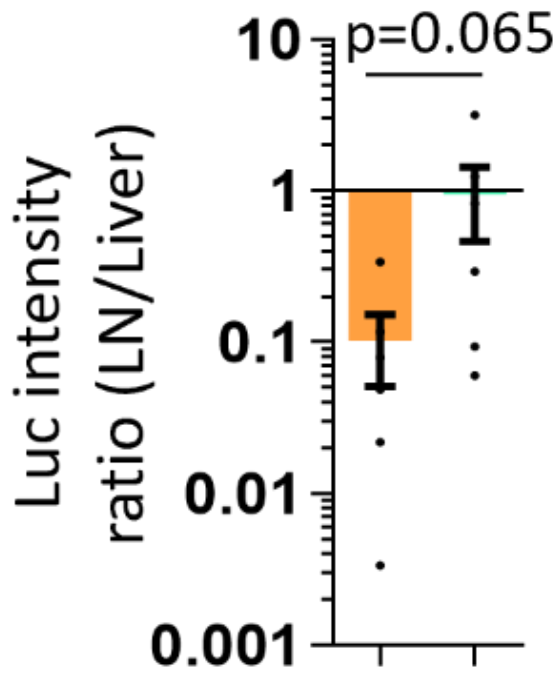

Supplementary Figure 12. The ratio of mRNA expression in the inguinal lymph node (LN) to the liver. Mice were injected with SM102-based CLNP and BM4-LNP, and lymph nodes were harvested 24 hours post-injection. Luciferase mRNA expression in the lymph nodes was normalized to luciferase expression in the liver (abdomen). Data are presented as mean ± S.E.M., mice n= 6 per group. The *P* = 0.065 was analyzed by the Mann-Whitney test.

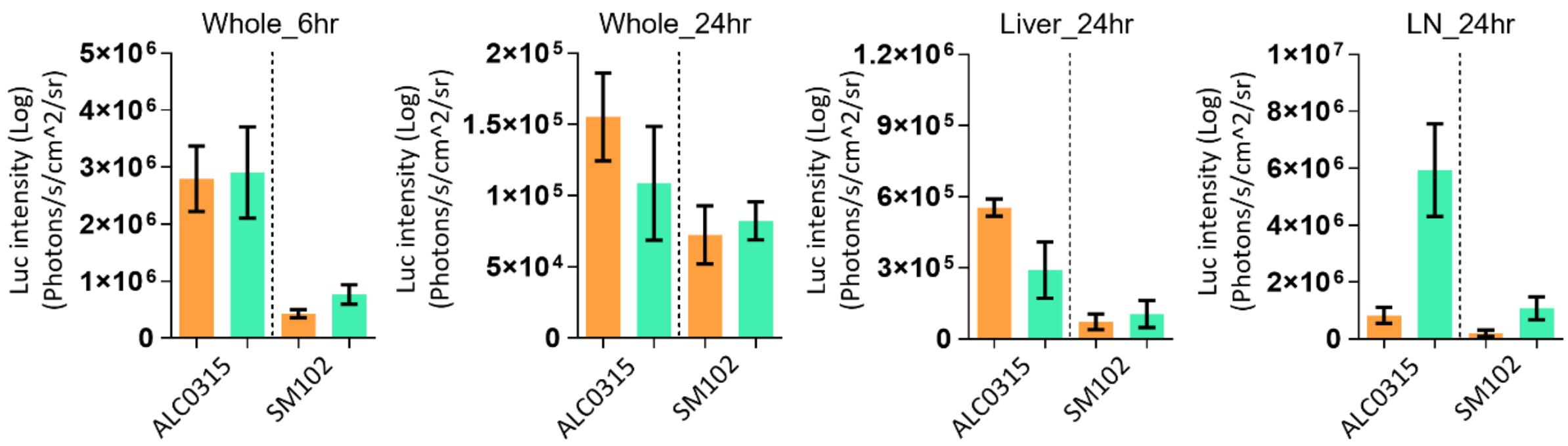

Supplementary Figure 13. Comparison of *in vivo* mRNA expression. mRNA expression levels of ALC0315- and SM102- LNPs were compared and shown. Data are presented as mean ± S.E.M., mice n= 4 for ALC0315 LNPs, and n= 6 for SM102 LNPs.

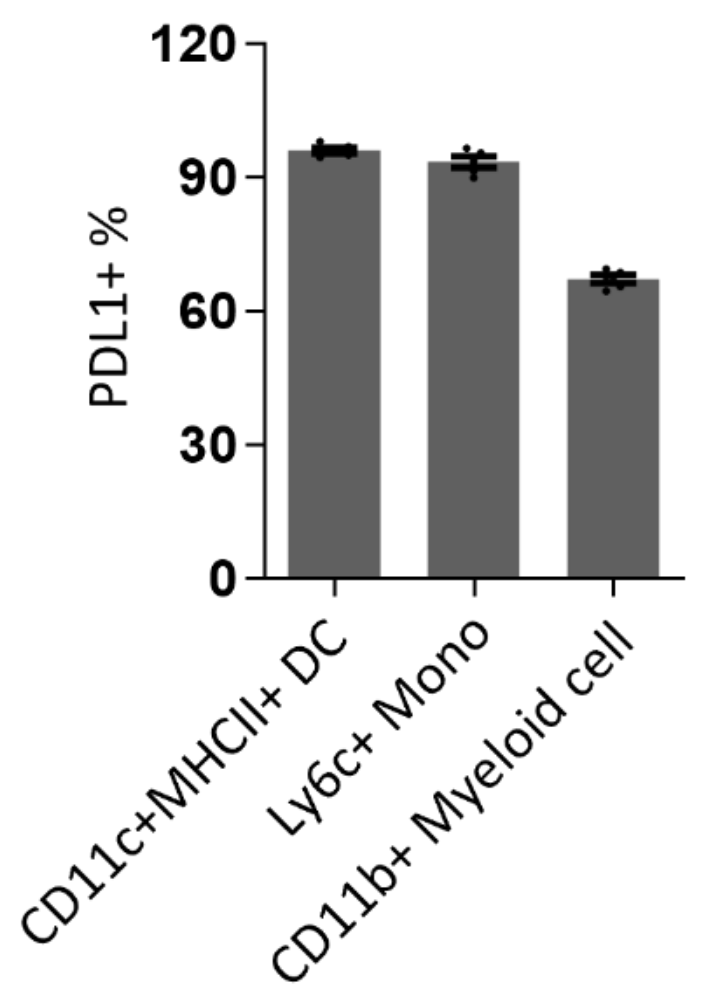

Supplementary Figure 14. PDL1 expression on immune cells in the lymph node. The ratio of PD-L1 expression on dendritic cells (DCs), inflammatory monocytes (Mo), and myeloid cells in the inguinal lymph node was analyzed using flow cytometry. Data are presented as mean ± S.D., mice n= 5.

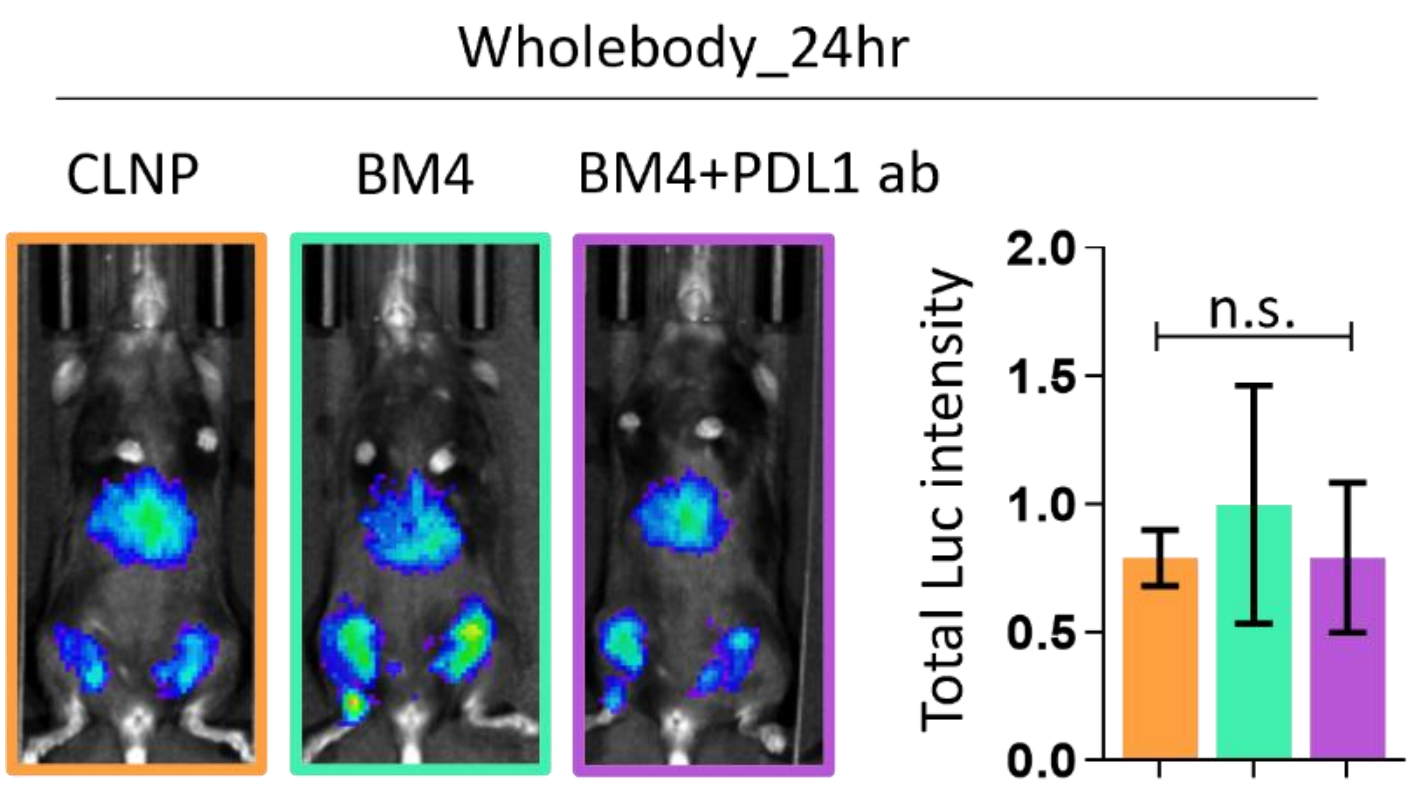

Supplementary Figure 15. PDL1 expression on immune cells in the lymph node. PDL1 blockade of BM4-LNP mRNA expression at the whole-body level. Data are presented as mean ± S.E.M., mice n= 3-4 per group. The n.s. was analyzed by one-way ANOVA with Tukey's post-test.

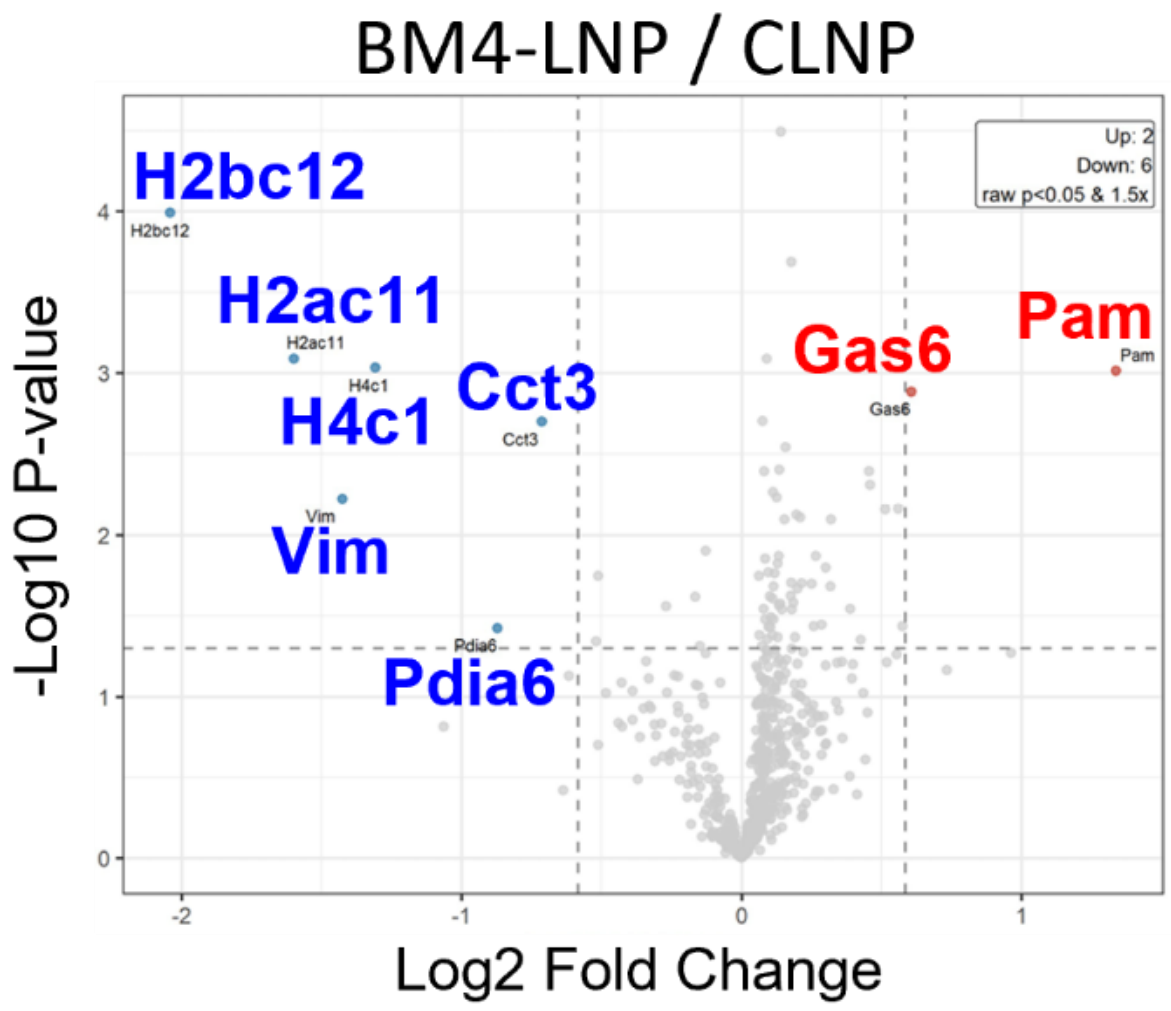

Supplementary Figure 16. Protein corona analysis of CLNP versus BM4-LNP. Eight proteins with statistical significance are shown. Growth arrest-specific protein 6(Gas6) and Peptidyl-glycine alpha-amidating monooxygenase (Pam) were upregulated, and Histone H2B type 1-F/J/L (H2bc12), Histone H2A type 1-B (H2ac11), Histone H4 (H4c1), T-complex protein 1 subunit gamma (Cct3), Protein disulfide-isomerase A6 (Pdia6), and Vim (Vimentin) were downregulated on BM4-LNP.

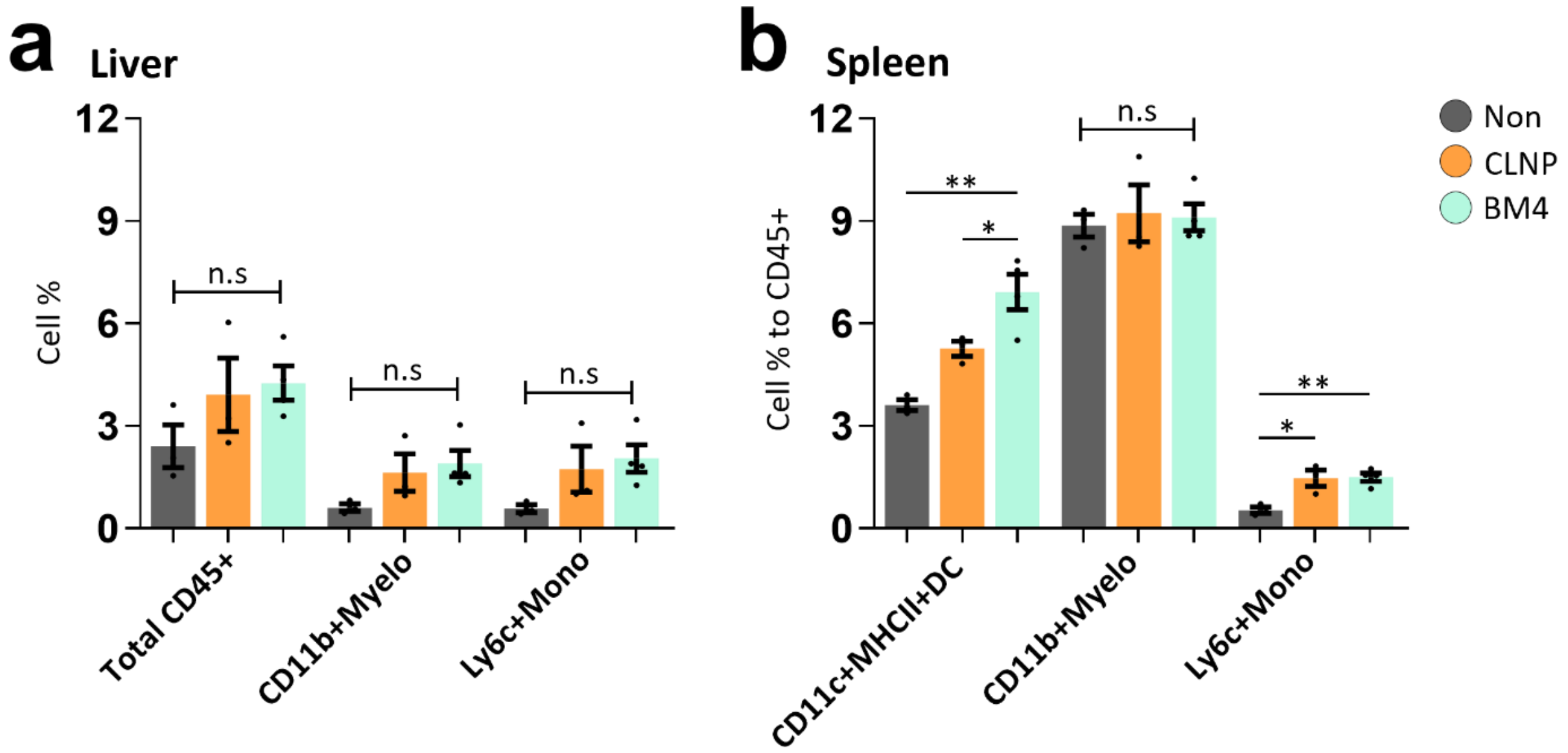

Supplementary Figure 17. Effects of LNPs on immune cells in the liver & spleen. At 24 hours post-LNP injection, the liver and spleen were harvested and analyzed by flow cytometry **(a)** Effect on hepatic immune cells. **(b)** Effect on splenic immune cells. Data are presented as mean ± S.E.M., mice n=3-4 per group. The **P*<0.05, ***P*<0.01, and n.s. were analyzed by one-way ANOVA with Tukey's post-test.
